# Digestive cancers: mechanisms, therapeutics and management

**DOI:** 10.1038/s41392-024-02097-4

**Published:** 2025-01-15

**Authors:** Tianzuo Zhan, Johannes Betge, Nadine Schulte, Lena Dreikhausen, Michael Hirth, Moying Li, Philip Weidner, Antonia Leipertz, Andreas Teufel, Matthias P. Ebert

**Affiliations:** 1 Department of Medicine II, University Medical Center Mannheim, Medical Faculty Mannheim, Heidelberg University, Mannheim, Germany; 2 DKFZ Hector Cancer Institute at University Medical Center Mannheim, Mannheim, Germany; 3 Mannheim Cancer Center, Medical Faculty Mannheim, Heidelberg University, Mannheim, Germany; 4 Molecular Medicine Partnership Unit, European Molecular Biology Laboratory, Heidelberg, Germany; 5 Junior Clinical Cooperation Unit Translational Gastrointestinal Oncology and Preclinical Models, German Cancer Research Center (DKFZ), Heidelberg, Germany

**Keywords:** Gastrointestinal cancer, Gastrointestinal cancer

## Abstract

Cancers of the digestive system are major contributors to global cancer-associated morbidity and mortality, accounting for 35% of annual cases of cancer deaths. The etiologies, molecular features, and therapeutic management of these cancer entities are highly heterogeneous and complex. Over the last decade, genomic and functional studies have provided unprecedented insights into the biology of digestive cancers, identifying genetic drivers of tumor progression and key interaction points of tumor cells with the immune system. This knowledge is continuously translated into novel treatment concepts and targets, which are dynamically reshaping the therapeutic landscape of these tumors. In this review, we provide a concise overview of the etiology and molecular pathology of the six most common cancers of the digestive system, including esophageal, gastric, biliary tract, pancreatic, hepatocellular, and colorectal cancers. We comprehensively describe the current stage-dependent pharmacological management of these malignancies, including chemo-, targeted, and immunotherapy. For each cancer entity, we provide an overview of recent therapeutic advancements and research progress. Finally, we describe how novel insights into tumor heterogeneity and immune evasion deepen our understanding of therapy resistance and provide an outlook on innovative therapeutic strategies that will shape the future management of digestive cancers, including CAR-T cell therapy, novel antibody-drug conjugates and targeted therapies.

## Introduction

Malignancies of the digestive system, including gastrointestinal and hepatobiliary cancers, are major contributors to global cancer morbidity and mortality. Colorectal and gastric adenocarcinomas are among the cancer entities with the highest global incidence. Together, the most prevalent digestive cancers, including colorectal cancer (CRC), esophageal cancer, gastric cancer (GC), hepatocellular carcinoma (HCC), and pancreatic ductal adenocarcinoma (PDAC) account for up to 33–35% of cancer-related deaths worldwide.^1,2^ CRC remains the most common digestive cancer in all regions of the world except Africa. Particularly in high-income countries, the introduction of complex multimodal therapies and surveillance programs has led to a significant reduction in CRC-related mortality over the past decades.^1,3,4^ Increasing incidence, particularly in developing countries, as well as a shift in the age at diagnosis with an increasing proportion of younger CRC patients pose new challenges in prevention and treatment for this cancer entity. A similar trend towards an increasing subgroup of younger patients has been observed for GC. At the same time, global incidence and mortality associated with GC have decreased in recent decades, either due to the implementation of screening programs in high-risk regions such as Korea or Japan^5,6^ or due to improvements in hygiene and treatment of *H. pylori* infection.^7^ However, on a global scale, early detection of gastric or gastroesophageal junction (GEJ) adenocarcinoma remains the exception and most cancers are diagnosed at an advanced stage with an overall high mortality. Together with the significant increase in the prevalence of obesity-related liver damage, particularly metabolically associated steatohepatitis (MASH), the incidence of HCC is rising significantly in high-income countries. In contrast, its incidence is declining in Asia due to improvements in primary prevention of viral hepatitis as the main etiology in this region.^1^ This regional and etiological shift may also affect therapeutic strategies due to the influence of altered metabolic and inflammatory states in MASH compared to hepatitis-related HCC. With 511,000 new cases of and 467,000 cancer-related deaths in 2022, pancreatic cancer remains one of the deadliest cancer entities^2^ and in the light of an aging society a rising incidence is expected.^8^ Improvements in imaging quality and accessibility will enhance early detection, and the introduction of neoadjuvant chemotherapy enables a larger subset of patients to be transferred to surgery. However, despite being only the twelfth most common cancer entity, PDAC remains the sixth most common cause of cancer-related mortality.^2^ This observation can be extrapolated to gastrointestinal and hepatobiliary cancers in general, which contribute to 1.4–1.8 times as many cancer deaths as they do to cancer cases due to difficulties in early detection, rendering curative treatment options unavailable at the time of diagnosis.^1^ The establishment of effective interventions that transfer advanced disease stages into potentially curative cases or enable a prolonged survival with good quality of life in non-curative situations remain, therefore, urgent clinical needs.

**Table 1:** Selection of ongoing clinical trials investigating novel immunotherapeutic interventions in metastatic digestive cancers

Cancer	Stage	Treatment line	Target	Treatment	Phase	Identifier	Status
ESCC	IV	≥2	PD-1 + HIF-2α + TKI	A: Pembrolizumab + Belzutifan + Lenvatinib B: Pembrolizumab + Lenvatinib	2	NCT04976634	Recruiting
Gastric/GEJ adenocarcinoma	IV or unresectable	Naive & ≥1	HER2; PD-1; PD-L1/CTLA-4; PD-1/TIGIT	Trastuzumab-Deruxtecan ± Pembrolizumab or Durvalumab or Volrustomig or Rilvegostomig ± Chemotherapy versus Trastuzumab + Chemotherapy	1b/2	NCT04379596	Recruiting
GEJ adenocarcinoma	IV or unresectable	≥2	TKI + PD-1	A: Regorafenib ± Nivolumab B: SOC	2b	NCT04879368	Active, not recruiting
CRC	IV; MSS	≥2	CTLA-4 + PD-1	A: Botensilimab and Balstilimab B: Botensilimab C: SOC	2	NCT05608044	Active, not recruiting
CRC	IV; pretreated, MSS, MSI-low	≥2	VEGFR, MET, TAM kinases ± PD-L1	A: Zanzalintinib + Atezolizumab B: Regorafenib	3	NCT05425940	Recruiting
CCA	I-III	Adjuvant	PD-L1 + CTLA-4	A: Capecitabin + Durvalumab + Tremelimumab B: Durvalumab + Tremelimumab	2	NCT05239169	Active, not recruiting
CCA	IV or unresectable	≥2	PD-1 + TKI	Durvalumab + Regorafenib	1/2	NCT04781192	Recruiting
PDAC*	IV	≥2	PD-L1 + SEMA4D	Avelumab and Pepinemab	1b/2	NCT05102721	Recruiting
PDAC	IV	Adjuvant	PD-L1 + Vaccine (mRNA)	A: Autogene Cevumeran + Atezolizumab + mFOLFIRINOX B: mFOLFIRINOX	2	NCT05968326	Recruiting
HCC	Unresectable, locoregional	I	PD-1 +VEGF	A: Durvalumab + TACE B: Durvalumab + Bevacizumab + TACE C: TACE	3	NCT03778957	Active, not recruiting
HCC	Resectable	I (perioperative)	PD-L1 + CTLA-4	Durvalumab + Tremilimumab	2	NCT05440864	Recruiting

*ESCC* esophageal squamous cell carcinoma, *GEJ* gastroesophageal junction, *PDAC* pancreatic ductal adenocarcinoma, *CRC* colorectal cancer, *CCA* cholangiocellular carcinoma, *HCC* hepatocellular carcinoma, *TKI* tyrosine kinase inhibitors, *HIF-2α* hypoxia inducible factor 2a, *MSS* microsatellite stability, *MSI* microsatellite instability, *TIGIT* T cell immunoreceptor with Ig and ITIM domains

Surgical resection remains the most common curative treatment for digestive cancers. Progress of surgical techniques, including robotic surgery, as well as improved perioperative management has reduced treatment-associated mortality and increased rates of curative resections.^9,10^ The introduction of multimodal therapeutic concepts, including neoadjuvant chemo(radio)therapy, or the combination of surgery, local ablative interventions and chemotherapeutic approaches, has further improved survival of many digestive cancers.^11^ Nevertheless, while a multimodal therapeutic approach appears feasible in locally advanced and even metastatic CRC, its utility in the management of other cancers of the digestive system remains the subject of ongoing scientific research. Over the past decades, there has been a significant advancement in our understanding of the molecular mechanisms underlying carcinogenesis, as well as the intricate interplay between cancer cells and the surrounding stroma and immune system, termed cancer microenvironment. The addition of monoclonal antibodies targeting human growth factor receptors, such as EGFR or HER2, to conventional chemotherapy has improved survival in patients with metastasized GC and CRC.^12–14^ However, the presence of activating mutations downstream of the targeted receptor or the lack of expression of the target renders this therapeutic approach inaccessible for most cancer patients. The advent of immune checkpoint inhibitors has enabled the implementation of a novel, highly effective therapy for most malignancies of the digestive tract. While very effective in patients with microsatellite instability, the response in other digestive cancers is less pronounced. While the combination of immune checkpoint inhibitors and inhibitors of angiogenesis has proven effective in the treatment of hepatocellular carcinoma^15^, the use of similar combinatorial therapies in cancers of the digestive system is still under evaluation. This rapid evolution of medical therapy has also improved the oncological outcomes for patients with rare cancers of the digestive system, such as neuroendocrine or gastrointestinal stromal tumors. The current therapeutic concepts and progress of these cancer entities are not presented in this work, but covered by excellent recent reviews.^16,17^

As a deeper understanding of tumor biology has led to the constant introduction of effective novel targeted immunotherapies for malignancies of the digestive system, cancer researchers and oncologists must be aware of the dynamically changing therapeutic landscape, both for optimizing oncological management and prioritizing research strategies. In this review, we provide a comprehensive overview of the epidemiology, molecular pathology, and current management of the six most common cancers of the digestive system, focusing primarily on the rapidly evolving field of pharmaco- and immunotherapy. Furthermore, we discuss challenges to current therapies and provide insights into novel therapeutic strategies that are pursued to improve the treatment of these cancer entities.

## Esophageal squamous cell carcinoma

### Epidemiology

With ~511,000 new cases and 445,000 deaths in 2022, esophageal cancer is the eleventh most frequently diagnosed cancer and the seventh leading cause of cancer-related mortality worldwide (GLOBOCAN 2022).^2^ The incidence and mortality of esophageal cancer in males are 2 to 3-fold higher than in females. Globally, esophageal cancer is most common in Eastern Asia, especially in China, and Eastern Africa, followed by Southern Africa, Northern Europe and South Central Asia.^2^ There are two main histologic subtypes of esophageal cancer, namely squamous cell carcinoma and adenocarcinoma. Esophageal SCC (ESCC) and esophageal adenocarcinoma show distinct differences in molecular characteristics: whereas ESCC resembles other SCCs such as head and neck cancer, esophageal adenocarcinoma exhibits a great similarity to chromosomal instability (CIN) gastric cancer.^18^ This section will mainly focus on ESCC, while adenocarcinoma of the esophagus, which frequently occurs at the gastroesophageal junction, will be discussed in the next chapter. The major risk factors for ESCC are tobacco smoking and overt alcohol consumption. The simultaneous consumption of cigarettes and alcohol synergistically increases the risk for ESCC 10 to 23.9 times compared to abstinent non-smokers.^19^ Further risk factors for ESCC include nutritional deficiency, betel quid chewing, intake of pickled vegetables or nitrosamine, and thermal injuries through consumption of hot food or beverages.^20^ Although ESCC represents approximately 90% of all esophageal cancer cases worldwide, the incidence rates of ESCC are declining in most countries, partly due to decreased levels of smoking as well as dietary changes.^1^

### Molecular pathology and tumor biology

ESCC develops from precursor lesions, namely squamous dysplasia presented with nuclear abnormalities, loss of normal cellular polarity, and aberrant tissue maturation in histology.^21^ The squamous dysplasia has a subtle macroscopic appearance and is easily missed when viewed by conventional white-light endoscopy.^22^ Typically, the oncogenesis of ESCC is initiated when esophageal mucosa is directly exposed to carcinogenic agents such as tobacco and alcohol, the main risk factors as mentioned above. The susceptibility is increased in case of mechanical injury such as in achalasia, after radiotherapy or burns with acids or alkalies.^23^ At the molecular level, ESCC is driven by both heritable and somatic DNA aberrations.^24^ Over the last decade, high-throughput genetic, proteomic, transcriptomic, and epigenetic analysis enabled a deeper understanding of the molecular biology of ESCC. A joint analysis of three genome-wide association studies identified several genetic susceptibility loci in the Chinese population, including novel loci in TMEM173 or the intronic region of ATP1B2.^25^ A study by the Cancer Genome Atlas Research Network performed whole-exome sequencing and single-nucleotide polymorphism (SNP) profiling of 90 ESCC tissues, and compared the results to 72 esophageal adenocarcinomas.^18^ At the gene expression level, ESCCs showed upregulation of Wnt, syndecan and p63 pathways which play important roles for squamous epithelial cell differentiation.^18^ In ESCC, the most frequent genetic mutations occur in *TP53, NFE2L2, MLL2, ZNF750, NOTCH1*, and *TGFBR2*.^13^ The most common copy number changes were identified in *SOX2, TERT, FGFR1, NKX2-1*, and deletions were observed in *RB1* and at the 3p25.2 locus.^18^ Also, frequent cell cycle dysregulation was observed based on combined data of genetic mutations and somatic copy number alterations.^18^ Furthermore, alterations of receptor tyrosine kinases and downstream signaling mediators, dysregulation of the TGF-β pathway, and alterations of chromatin-remodeling were identified in ESCC.^18^ Three molecular subclasses of ESCC were proposed, but the clinical impact of this classification is yet unclear.^18^

### Current management

The therapy of ESCC is based on tumor stage, location, and patients´ functional status and comorbidities.^20^ Also, nutritional support should be provided in patients with ESCC, as weight loss is common and associated with increased operative risk, deteriorating quality of life, and poor survival in advanced disease.^20^

### Management of early-stage ESCC

Endoscopic en bloc resection is the treatment of choice for most early-stage ESCC (cT1N0M0) according to European Society for Medical Oncology (ESMO) recommendations.^20^ Superficial ESCC cancer can be resected by endoscopic mucosal resection or endoscopic submucosal dissection. Endoscopic mucosal resection involves lifting of the target lesion by fluid injection, followed by direct snare excision or cap-assisted suction and resection.^22^ Endoscopic submucosal dissection is based on circumferential cutting and dissection of submucosal tissues underneath the target lesion.^22^ Although being technically more difficult and considerably more time-consuming than endoscopic mucosal resection^22^, submucosal dissection overcomes the problem of piecemeal resections even in large lesions^26^ and demonstrates a higher rate of en bloc resection (100 versus 53.3%) and a lower local recurrence rate (0.9 versus 9.8%) in early ESCC.^22^ Large Asian studies reported a high rate of 5-year survival (90–92%) in patients with superficial esophageal squamous cell neoplasia undergoing endoscopic submucosal dissection.^22,23^ Beyond the aforementioned resection techniques, endoscopic ablation techniques can destroy neoplastic tissues through thermal injury. As such, argon plasma coagulation and radiofrequency ablation cause immediate coagulation necrosis, whereas cryotherapy causes immediate and delayed tissue injury through freezing.^22^ However, the role of ablation therapy in the treatment of early ESCC is not established.^22^ Notably, lymph node metastases occur early and represent the most important prognostic factor in ESCC.^27^ Therefore, patients with deep intramucosal (T1a, sm3) and submucosal tumors (T1b), surgical tumor resection with lymphadenectomy, or chemoradiation with organ preservation should be offered as the preferred treatment options.^20^

### Management of locally advanced, resectable ESCC

The treatment of locally advanced, resectable ESCC (cT2-T4 or cN1-3 M0) requires multidisciplinary management. Neoadjuvant chemoradiotherapy followed by surgery is the current standard based on the results of the phase III CROSS trial.^28^ In this trial, weekly carboplatin/paclitaxel and concurrent radiation of 41.4 Gy in 23 fractions followed by esophagectomy showed a significant survival advantage compared to surgery alone, without increasing postoperative morbidity and mortality in patients with esophageal cancer.^20^ In the trimodality arm, the response rate in ESCC is high with 49% patients showing pathological complete response, and 5-year survival in patients with ESCC was over 50%.^28^ Until recently, adjuvant therapy following trimodal therapy to reduce disease recurrence has not been established. The phase III trial CheckMate 577 demonstrated that patients with residual pathological disease of esophageal or gastroesophageal junction cancers (≥ypT1 and/or ≥ypN1) significantly benefited from postoperative treatment with the immune checkpoint inhibitor nivolumab compared to placebo (median disease-free survival 22.4 versus 11.0 months).^29^ These results have led to the approval of nivolumab as adjuvant therapy in patients with esophageal cancer, showing non-pathological complete response following neoadjuvant chemoradiation and surgery. The alternative to trimodality therapy is definitive chemoradiation with close surveillance and the option of salvage esophagectomy in selected cases^20^, especially for those who are unfit for surgery or suffer from cervical ESCC. Randomized trials demonstrated non-inferior overall survival in patients treated with definitive chemoradiation compared to those treated with chemoradiation and surgery.^29,30^ However, in those with clinical non-response to chemoradiation, the overall survival of non-operated patients was significantly worse.^31^ A further large retrospective analysis revealed that the overall survival in patients showing persistent or recurrent disease after definitive chemoradiation treated with salvage esophagectomy did not differ from the overall survival of patients receiving chemoradiation and planned surgery, underlining the value of salvage surgery.^32^ Most definitive chemoradiation regimens consist of fluorouracil-platinum combination chemotherapy with a higher dose of concurrent radiation (50–66 Gy).^29–35^

### Management of unresectable or metastatic ESCC

**Fig. 1:**
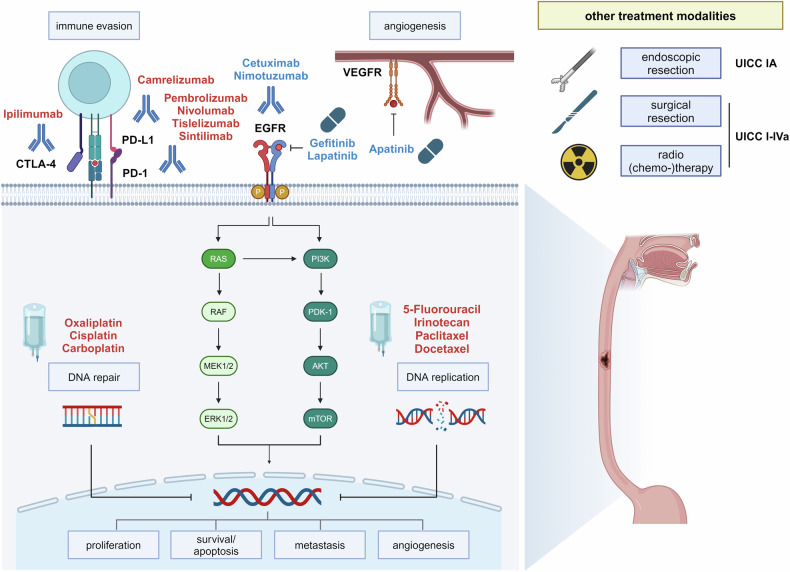
Current therapeutic targets in esophageal squamous cell carcinoma (ESCC). 5-FU 5-fluorouracil, PD-1 programmed cell death protein 1, PD-L1 programmed cell death 1 ligand 1, CTLA-4 cytotoxic T-lymphocyte-associated protein 4, EGFR epidermal growth factor receptor, VEGFR vascular endothelial growth factor receptor. Therapies that are recommended by international guidelines are marked in red. Therapies with preclinical or only low levels of clinical evidence are highlighted in blue

The treatment of advanced unresectable or metastatic ESCC has been limited to chemotherapy with a poor prognosis. In recent years, ESCC was found to respond to immune checkpoint inhibitors, demonstrating survival benefits and therefore significantly changing the current therapeutic algorithms (see also Fig. 1).^36–42^ Through blockade of the programmed death receptor 1 (PD-1) on cytotoxic T-cells, its ligand (PD-L1) on tumor cells, or CTLA-4 receptor on T-cells, cancer cell-mediated immune escape could be downregulated and anti-tumor immune response could be promoted. The expression level of PD-L1 is a predictive biomarker for treatment with immune checkpoint inhibitors in esophageal and gastric cancers. Immunohistochemistry is used to assess PD-L1 expression through the tumor proportion score (TPS) and the combined positivity score (CPS). In clinical practice, PD-L1 expression, as well as MMR/ MSI status should be determined in unresectable or metastatic ESCC before therapy initiation.

First-line therapy for advanced or metastatic ESCC has been chemotherapy with fluoropyrimidine plus platinum in Western countries and paclitaxel plus platinum in Asia.^33^ The median overall survival was reported to be less than one year.^36^ Immune checkpoint inhibitors have emerged as new therapy options based on evidence from several multicenter, randomized phase III studies. In the KEYNOTE-590 trial, first-line therapy with PD-1 antibody pembrolizumab plus 5-FU/cisplatin-based chemotherapy significantly improved overall survival compared to chemotherapy alone in patients with esophageal or gastroesophageal junction cancer. This survival benefit was most pronounced in patients with ESCC and PD-L1 CPS ≥10 (median overall survival 13.9 versus 8.8 months), whereas there was no significant benefit observed in patients with CPS <10.^37^ These results led to approval of pembrolizumab in the first-line treatment of locally advanced or metastatic ESCC, adenocarcinoma of the esophagus and GEJ adenocarcinoma with high PD-L1 expression (CPS ≥10). The CheckMate 648 study compared first-line therapy of anti-PD-1-antibody nivolumab plus chemotherapy or plus anti-CTLA-4 antibody ipilimumab to chemotherapy alone in patients with advanced or metastatic ESCC. In patients with PD-L1 expression of 1% or higher, nivolumab plus chemotherapy yielded a superior median overall survival compared to chemotherapy alone (15.1 versus 9.1 months). In those patients, the therapy regimen of nivolumab plus ipilimumab also offered a survival advantage over chemotherapy alone (median overall survival 13.7 versus 9.1 months). These data led to the approval of nivolumab plus fluoropyrimidine- and platinum-containing chemotherapy and nivolumab plus ipilimumab for first-line treatment of patients with advanced or metastatic ESCC and a PD-L1 expression of 1% or higher. Furthermore, the ESCORT-1, ORIENT-15 and JUPITER-06 trials showed that the anti-PD-1 antibodies camrelizumab, sintilimab and toripalimab prolonged overall survival when added to chemotherapy in Asian patient populations.^38–40^ Immunotherapy has also changed the landscape of second-line treatment of advanced or metastatic ESCC, which had only a few options previously. The phase III ATTRACTION-3 trial showed a significant survival advantage of second-line nivolumab monotherapy compared to taxane-based chemotherapy in patients with advanced or recurrent ESCC regardless of PD-L1 expression (10.9 versus 8.4 months), with the majority of patients from Asia.^41^ In the multicenter, phase II RAMONA trial enrolling patients aged ≥65 years with advanced ESCC in Germany, nivolumab with or without ipilimumab as second-line treatment was safe and beneficial.^42^ In the phase III KEYNOTE-181 trial, second-line pembrolizumab monotherapy prolonged overall survival in patients with advanced ESCC and a CPS ≥10 compared to chemotherapy (8.2 versus 7.1 months).^43^ Similarly, further anti-PD-1 antibodies tislelizumab and camrelizumab could improve overall survival compared to second-line chemotherapy.^44,45^ For subsequent lines, chemotherapy with taxane, rechallenge with platinum or irinotecan based chemotherapy may be considered.^20,46^

### Clinical research progress

With the encouraging results consistently reported by many phase III trials, immunotherapy has revolutionized the therapy of ESCC and become an integral part of the adjuvant as well as in the first- and second-line treatment of advanced or metastatic ESCC. Beyond the combination with chemotherapy, a novel combination strategy with tyrosine kinase inhibitors (TKI) is being tested. Currently, the randomized, phase III trial LEAP 014 evaluates the efficacy and safety of first-line lenvatinib plus pembrolizumab plus chemotherapy compared to pembrolizumab plus chemotherapy in patients with metastatic ESCC.^47^

Real-world data for immunotherapy in ESCC is also emerging. In most of the aforementioned clinical trials, only patients with good performance status (Eastern Cooperative Oncology Group [ECOG] 0-1) were enrolled, which led to a selection of younger and fit patients. However, in clinical practice, patients with ESCC often present with severe comorbidities and limited functionality. Although immune checkpoint inhibitors are generally better tolerated than conventional chemotherapy, they still exhibit their own toxicity profile.^48^ Severe immune-related adverse events including pneumonitis, hepatitis and colitis were observed in 4.8, 1.9, and 0.9% patients treated with pembrolizumab in the KEYNOTE-181 trial.^43^ It is further unclear how to predict the individual response to the recommended immunotherapy. PD-L1 expression, either measured by the TPS or CPS score, was shown to predict treatment efficacy.^37,41,49^ However, further predictive factors are needed. Another clinical challenge is to identify resistance mechanisms to immune checkpoint inhibitors. Using longitudinal molecular studies on tumor tissues or liquid biopsies during immunotherapy could help to understand tumor evolution and resistance development. Also, evidence needs to be generated whether treatment beyond progression or a re-exposure to immune checkpoint inhibitors is beneficial.

A further important area of interest is the definition of clinical complete response and the development of subsequent active surveillance strategies in patients with locally advanced ESCC after neoadjuvant chemoradiation, considering the high rate of pathological complete response (49%) reported in the CROSS trial.^28^ In the treatment of locally advanced rectal cancer, there is a paradigm shift towards a watch-and-wait strategy for patients showing clinical complete response after chemoradiation, despite the fact that pathological complete response rates in rectal cancer are much lower compared to ESCC. With an active surveillance strategy, patients could be spared from surgery and postoperative complications. However, accurate diagnosis of clinical complete response is pivotal to avoid false negative cases. Retrospective meta-analysis shows that overall survival may not be different between patients who achieved clinical complete response and underwent active surveillance, compared to those who received surgery.^50,51^ However, prospective studies comparing an active surveillance strategy versus surgery in patients with locally advanced ESCC responding to chemoradiation are needed and have been initiated.^52^

## Gastric and gastroesophageal junction adenocarcinoma

### Epidemiology

Gastric cancer (GC) is the fifth most common cause of cancer-related death worldwide, with an estimated 968.000 newly diagnosed cases in 2022, according to the most recent GLOBOCAN data.^2^ There is a significant sex-dependent difference in GC incidence with an age-standardized incidence rate of 12.8/100,000 in males and 6.0/100,000 in females.^2^ Incidence is highest in Asia followed by Europe and Latin America. Over the last decades the overall incidence of GC steadily decreased whereas it is increasing for GEJ adenocarcinoma.^53^ GC is generally uncommon in adults under 50, with incidence rates rising with age.^54^ Nevertheless, the incidence of GC among younger individuals is increasing in both high- and low-incidence regions, such as the United States, while in the elderly population, the overall incidence is declining.^55^ Due to subtle early symptoms of GC, most patients are diagnosed at an advanced or metastatic stage.^56^ Survival time for GC is closely linked to the tumor stage at diagnosis.^57^ Five-year relative survival in Japan was 81.0%, compared to 45.0% in the United States. This can be largely explained by differences in tumor stage at diagnosis, as many tumors are detected during screening for GC in Japan.^58^

### Molecular pathology and tumor biology

Persistent mucosal inflammation is the primary driver of carcinogenesis, leading to sporadic gastric neoplasia. The Kyoto Classification proposes environmental and host-related factors as causes of gastritis.^59^ Environmental factors, both transmissible such as *Helicobacter pylori* infection, and non-transmissible, are more common. In contrast, host-related factors are less frequent and often involve immune-mediated disorders. Both factors result in atrophic remodeling of the gastric mucosa and chronic immune stimulation, promoting epithelial neoplastic lesions via the inflammation-dysplasia-carcinoma sequence.^60^ While most *H. pylori*-infected individuals remain asymptomatic, nearly all show chronic gastric inflammation. Of those, about 10% develop peptic ulcer disease, 1–3% progress to GC, and 0.1% develop mucosa-associated lymphoid tissue (MALT) lymphoma.^61^ While *H. pylori* infection is one of the most important causes of GC, most infected individuals do not develop cancer. This may be due to the heterogeneity of the bacterial genome and its virulence factors such as the CagA oncoprotein or BaB A and SAbA adhesins.^62^ CagA interacts with various signaling pathways in the gastric epithelium, destabilizing cellular junctions, and activating pro-inflammatory and oncogenic pathways. This leads to disturbances in the integrity, differentiation, and self-renewal of the gastric epithelium. Other mechanisms by which *H. pylori* infection drives gastric carcinogenesis include degradation of the tumor suppressors p14ARF^63^, disruption of the gastric mucosal barrier, and promotion of epithelial-to-mesenchymal transition (EMT).^64–66^ Furthermore, CagA+ *H. pylori* infection dysregulates the Hippo signaling pathway, responsible for the control of stem cell properties and proliferation, thereby promoting EMT and tumorigenesis.^67,68^ *H. pylori* strains not only alter host mechanisms, but also undergo genetic modifications in carcinogenic environments, with certain strains associated with premalignant lesions.^69^ Recently, *Streptococcus anginosus*, a resident bacteria of the oral cavity and gastrointestinal tract, was shown to drive gastric carcinogenesis in murine models.^70^ *S. anginosus* is enriched in GC tissues and directly interacts with gastric epithelial cells by disrupting tight junctions and inducing MAPK signaling. Beyond the impact of bacterial pathogens, host and environmental factors shape individual risks to develop GC. Proximal GC and GEJ adenocarcinoma are linked to gastroesophageal reflux disease and the formation of Barrett’s mucosa.^71^ About 10% of all GC exhibits underlying genetic predispositions.^71^ Genes associated with hereditary GC include, for instance, *CDH1* (E-cadherin), *CTNNA1*^72,73^, *FBXO24*, or *DOT1L*.^74^ GC can also occur in association with other well-defined hereditary cancer syndromes, such as familial adenomatous polyposis, Cowden syndrome, Lynch syndrome, MUTYH-associated adenomatous polyposis or Li-Fraumeni syndrome.^75^

GC is commonly classified by its histomorphology, but additional molecular classifications have been proposed. The Laurén classification is the most frequently used classifier for GC and divides cancers into intestinal, diffuse, and intermediate subtypes depending on their histological phenotype.^76^ Based on molecular differences, the Cancer Genome Atlas Research Network proposed a classification dividing GC into 4 molecular subgroups: microsatellite instable (MSI), Epstein Barr Virus-positive (EBV), genomically stable (GS) and chromosomal instability (CIN).^77^ Microsatellite instability in GC (MSI‑GC) is characterized by a high mutational load and CpG-island methylator phenotype, with MLH1 frequently silenced in these tumors. Based on literature, the frequency of MSI-GC varies significantly between 0–44.5%.^70^ MSI-GC often expresses PD-L1, which is an important predictive marker for immune checkpoint inhibitor therapy.^78^ Epstein Barr virus‑associated GC (EBVaGC) accounts for 2–20% of all GC^70^ and shows a CpG-island methylator phenotype similar to MSI-GC. However, EBVaGC and MSI-GC are mutually exclusive.^79^ EBVaGC is more common among Caucasians, in male and younger patients. This subtype can also express PD-L1 and may respond to immune checkpoint inhibitors.^80,81^ Chromosomal unstable GC (CIN GC) are characterized by frequent mutations in the tumor suppressor gene TP53, which is also the most frequently altered gene in all GC.^77^ CIN GC often contains amplifications of genes encoding plasma-membrane bound receptors that present actionable targets, such as EGFR, FGFR2, HER2, and MET.^82^ Genomically stable GCs predominantly show a diffuse histological phenotype. In addition to *CDH1* and *RHOA* mutations, rearrangements between *CLDN18* and *ARHGAP26*, or *ARHGAP6* are also observed in this subtype. Beside the Cancer Genome Atlas Research Network, the Asian Cancer Research Group also proposed a four-group classification based on mRNA expression, somatic copy number alterations and gene mutations, which is also associated with specific histological phenotypes and clinical prognosis.^83^ However, these two molecular classifiers are based on multi-omics profiling of GC and not currently used for therapy decisions in clinical practice.

### Current management

#### Management of resectable GC and GEJ adenocarcinoma

##### Perioperative therapy

While endoscopic resection can be performed in early GCs, for the majority of localized GC and GEJ adenocarcinoma, surgical resection remains the main curative treatment option in more advanced, non-metastatic stages. In this context, perioperative chemotherapy has become the standard treatment for resectable, localized GC and GEJ adenocarcinoma. There are several clinical trials demonstrating improvement of oncological outcomes with perioperative chemotherapy. The phase II/III FLOT4-AIO study compared perioperative FLOT (fluorouracil, leucovorin, oxaliplatin, and docetaxel) with ECF/ECX (epirubicin, cisplatin, and 5- fluorouracil/capecitabine) regimen.^84^ FLOT chemotherapy improved overall survival (50 versus 35 months) and is currently the standard regimen for perioperative treatment in many countries. Recently, the results of the German ESOPEC trial showed that the perioperative FLOT regimen is superior to neoadjuvant CROSS radiochemotherapy in patients with GEJ adenocarcinoma, with an overall survival benefit of 66 versus 37 months.^85^ Most clinical trials evaluating perioperative chemotherapy were conducted in Western countries, while this therapeutic approach is less common in Asia. However, two important trials with Asian cohorts also confirmed that perioperative chemotherapy is superior to adjuvant therapy. In the phase III PRODIGY trial, patients received either neoadjuvant treatment with docetaxel, oxaliplatin, and S-1, followed by surgery and S-1 adjuvant chemotherapy.^86^ For the adjuvant treatment arm, patients received upfront radical surgery followed by S-1 chemotherapy. The results of this trial showed that perioperative treatment improved progression-free survival. The Chinese RESOLVE trial compared perioperative S-1 and oxaliplatin versus primary resection and adjuvant therapy (SOX or CAPOX), also showing superior oncological outcomes for perioperative treatment.^86^

Targeted therapies against HER2 and VEGF are standard treatment options in metastatic GC. However, their role in the perioperative or adjuvant setting, in combination with chemotherapy, is less clear and currently under investigation. For HER2 positive GC, the single-arm HER-FLOT study investigated the combination of trastuzumab and FLOT as perioperative treatment for locally advanced GEJ adenocarcinoma.^87^ The primary endpoint of achieving a pathological response rate >20% was reached by the study. The PETRARCA trial assessed the efficacy of adding trastuzumab and pertuzumab to perioperative FLOT chemotherapy in patients with HER2-positive GC and was prematurely closed after the same antibody combination did not meet its primary endpoint in a trial with metastatic GC.^88^ However, the pathological complete response rate was also significantly improved with this combination therapy. Despite the positive effects on remission rates, HER2-targeted therapy is currently not a standard option in perioperative treatment. Several trials evaluated the efficiency of anti-VEGF therapy in the perioperative management of GC. The ST03 trial assigned patients with GEJ adenocarcinoma to perioperative chemotherapy (ECX) with or without bevacizumab.^89^ The RAMSES/FLOT7 trial investigated the efficacy of ramucirumab in combination with FLOT.^87^ No differences in patient survival were observed in both studies. However, wound healing and postoperative complications were more prevalent in the anti-VEGF therapy group. Perioperative immune checkpoint therapy in combination with chemotherapy is currently being investigated by several studies. The MATTERHORN trial examines neoadjuvant durvalumab and FLOT chemotherapy followed by adjuvant durvalumab monotherapy in patients with resectable GC/GEJ adenocarcinoma.^88^ The DANTE trial combines perioperative FLOT chemotherapy with atezolizumab, while the KEYNOTE-585 trial assesses the addition of pembrolizumab to perioperative chemotherapy. Preliminary results from the DANTE trial showed higher rates of pathological complete response with addition of immune checkpoint inhibitors, particularly for MSI or PD-L1 expressing tumors.^90^ In the KEYNOTE-585 trial, that was a trend towards a longer progression-free survival for pembrolizumab treatment.^91,92^ Based on the current level of evidence, there is not yet a recommendation to combine perioperative chemotherapy with any targeted agents.

##### Adjuvant chemotherapy

Adjuvant chemotherapy is often used in Asia for patients who underwent primary resection with UICC stage II or III disease. The ACTS-GC trial from Japan assigned patients to receive either S-1 monotherapy after surgery or surgery alone.^93^ Results of the trial showed that adjuvant S-1 monotherapy results in an improved 3-year overall survival. The CLASSIC trial compared surgery followed by CAPOX adjuvant chemotherapy versus gastrectomy alone in patients with stage II-IIIB GC. Estimated 5-year disease-free survival was 68% in the adjuvant therapy versus 53% in the observation group.^94^ There is no standard chemotherapy regimen for adjuvant therapy. S1, SOX, or CAPOX are commonly used. Notably, patients with MSI-H/dMMR GC might benefit less from adjuvant therapy, as a meta-analysis indicates^95^, therefore it is not recommended for this subgroup of patients by the ESMO guidelines.^20^

#### Management of unresectable and metastatic GC and GEJ adenocarcinoma

**Fig. 2:**
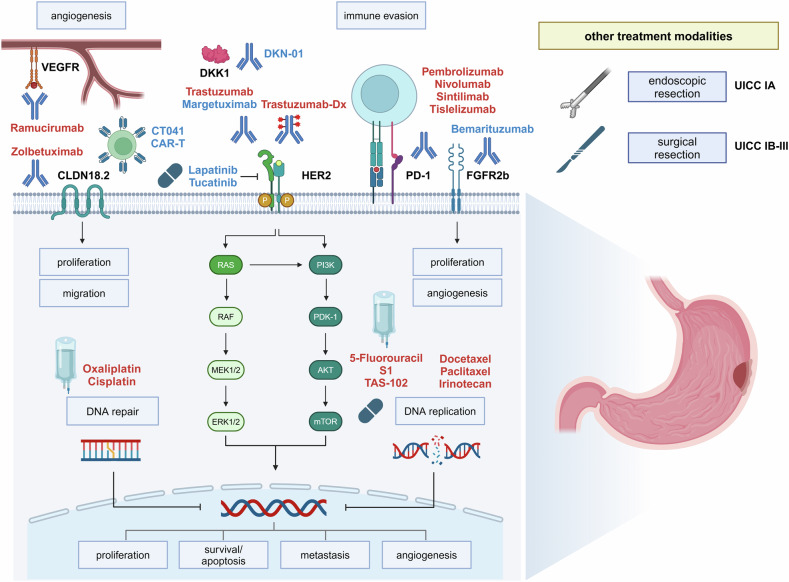
Current therapeutic targets in gastric cancer and GEJ adenocarcinoma. 5-FU 5-fluorouracil, VEGFR vascular endothelial growth factor receptor, HER2 human epidermal growth factor receptor 2, CLDN18.2 claudin 18.2, PD-1 programmed cell death protein 1, FGFR2b fibroblast growth factor receptor 2b, DKK1 Dickkopf WNT signaling pathway inhibitor 1. Therapies that are recommended by international guidelines are marked in red. Therapies with preclinical or only low levels of clinical evidence are highlighted in blue

First- and second-line therapy of metastatic GC and GEJ adenocarcinoma are based on a combination of chemotherapy and antibody therapy. Selection of therapy regimen largely depends on the performance status of the patient, prior therapies, and expression of predictive biomarkers, including HER2, MSI, PD-L1, and more recently CLDN18.2 (see Fig. 2). In clinical practice, it is recommended to determine the expression of these biomarkers in metastatic GC and GEJ adenocarcinoma, while the benefit of cancer genome sequencing for GC is less clear and should be limited to selected cases.

##### Chemotherapy

Although there is no standard first-line chemotherapy, a two-drug regimen of fluoropyrimidine and platinum is typically chosen for most patients. Oxaliplatin is generally preferred over cisplatin due to its lower toxicity and comparable effectiveness.^93^ Docetaxel-based triplet chemotherapies showed higher response rates and longer progression-free survival, but these advantages are associated with increased toxicity.^96^ Results from the phase III GASTFOX-PRODIGE 51 study evaluated adding docetaxel to FOLFOX in the first-line treatment of HER2-negative, advanced gastric or GEJ adenocarcinoma. The results demonstrated a significant improvement in oncological outcomes, with longer overall survival rates. However, the frequency of grade 3 toxicities was also higher in the triplet therapy group. Hence, triple therapy might be an option for patients with HER2, PD-L1, and CLDN18.2 negative tumors and a good performance status.^97^

##### Metastatic HER2-positive gastric cancers

In 2010, the European Medicines Agency approved trastuzumab for the first-line treatment of HER2-positive advanced gastric or GEJ adenocarcinoma. The ToGA trial showed that in patients with advanced HER2-positive GC, trastuzumab combined with platinum-based chemotherapy increased median overall survival to 13.8 versus 11.1 months with chemotherapy alone.^13^ HER2 testing thus became the first predictive biomarker for GC.^98^ Assessment of HER2 expression in GC is complex and requires specific training. HER2-positive resection specimens are defined by ≥10% tumor cells showing moderate 2+ immunostaining, with confirmation of HER2 amplification by FISH, or strong immunostaining (3+). Experts recommend at least five tumor-bearing biopsies to account for HER2 heterogeneity. The VARIANZ study highlighted the challenge of accurate HER2 assessment^99^, as it showed a high deviation between results from central and local assessments of HER expression, which also affected treatment outcomes. For the second-line therapy of HER2-positive metastatic GC, the antibody-drug conjugate trastuzumab-deruxtecan is currently approved. In the DESTINY-Gastric01 trial, trastuzumab-deruxtecan was compared to chemotherapy in patients with HER2-positive advanced GC and GEJ adenocarcinoma from Japan and South Korea.^100^ Overall survival was longer with trastuzumab-deruxtecan than with chemotherapy (12.5 versus 8.4 months), leading to the approval of the drug. Similar findings were observed in the follow-up DESTINY-Gastric02 trial which included patients from the USA and Europe.^99^

##### Metastatic PD-L1 positive gastric cancers

PD-L1 is expressed by various immune cells, such as lymphocytes and dendritic cells, and is often aberrantly expressed on solid tumors. PD-L1 binds to PD-1 on activated T-cells, suppressing T-cell receptor signaling and enabling immune evasion of tumors.^101^ Inhibiting the PD-1/PD-L1 interaction with checkpoint inhibitors can restore the immune response against cancer cells.^102^ PD-L1 is frequently expressed in GC and high expression is more common in men, tumors of the proximal stomach, and specific subtypes such as HER2-positive GC, EBVaGC or MSI-GC.^103^ Over the last years, several clinical trials have highlighted the clinical benefit of adding immune checkpoint inhibitors to chemotherapy in metastatic GC and GEJ adenocarcinoma. The CheckMate 649 trial investigated the efficacy of the anti-PD-1 antibody nivolumab and anti-CTLA-4 antibody ipilimumab in patients with advanced HER2-negative GC.^104^ A total of 1.581 previously untreated patients were randomized to receive either nivolumab plus chemotherapy (capecitabine with oxaliplatin or fluorouracil with leucovorin and oxaliplatin), chemotherapy alone, or nivolumab plus ipilimumab. Adding nivolumab to standard chemotherapy resulted in a statistically significant improvement in overall survival, with the most significant benefits observed in patients with a high CPS score. Similarly, the KEYNOTE-590 study demonstrated that pembrolizumab, another anti-PD-1 antibody, plus chemotherapy improved overall survival in advanced esophageal and GEJ adenocarcinoma compared to chemotherapy alone in patients with high CPS scores.^37^ These results were corroborated by the KEYNOTE-859 study which focused on locally advanced or metastatic HER2-negative gastric or GEJ adenocarcinoma.^105^ In this study, overall survival was higher in patients with high CPS scores who received pembrolizumab. Recently, the KEYNOTE-811 trial demonstrated that pembrolizumab can be combined with chemotherapy and trastuzumab in advanced or metastatic HER2-positive GC or GEJ adenocarcinoma, resulting in longer overall survival compared to chemotherapy and trastuzumab alone, which however did not reach the prespecified criteria for significance in the interim analysis.^106^ Based on the results of these trials, pembrolizumab and nivolumab were approved for the first-line therapy of HER2-negative and CPS-positive cancers in combination with fluoropyrimidine- and platinum-based chemotherapy.

##### Metastatic Claudin 18.2 positive gastric cancers

Claudin 18.2 (CLDN18.2) is part of the claudin family, which is crucial for the formation of paracellular barriers via tight junctions.^107^ The claudin 18.2 isoform is predominantly expressed in differentiated epithelial cells of the gastric mucosa and primary GC. The reported frequency of moderate or strong CLDN18.2 expression in GC varies between 17.4–52% and is not correlated with specific clinical features or histological phenotypes.^108–110^ However, these varying results might be due to different antibodies, staining protocols, and scoring systems. Ongoing clinical trials are expected to harmonize these methods. In the FAST trial, the chimeric monoclonal CLDN18.2 targeting antibody zolbetuximab was combined with first-line chemotherapy (epirubicin, oxaliplatin, capecitabine) in patients with advanced GC and GEJ adenocarcinomas with moderate-to-strong CLDN18.2 expression in ≥40% of tumor cells.^111^ This combination significantly improved progression-free and overall survival compared to chemotherapy alone. The benefit was even greater in patients with >70% of tumor cells expressing CLDN18.2. These findings were corroborated in the SPOTLIGHT trial that investigated the efficacy and safety of first-line zolbetuximab plus mFOLFOX6 versus chemotherapy alone in patients with CLDN18.2-positive, HER2-negative advanced or metastatic GC or GEJ adenocarcinoma.^112^ Zolbetuximab treatment showed a significant reduction in the risk of disease progression or death compared with placebo. The median progression-free survival was 10.6 months in the zolbetuximab versus 8.67 months in the placebo group. Similar outcomes for progression-free and overall survival were also shown for zolbetuximab plus CAPOX in the phase III GLOW trial.^113^ Based on these trials, approval of zolbetuximab for CLDN18.2-positive GC is expected soon.

##### Other molecular targets in metastatic gastric cancers

VEGF is a key cytokine involved in initiating tumor angiogenesis by stimulating endothelial cell growth and enhancing vascular permeability. Elevated VEGF expression is frequently observed in gastric cancer tissues and correlates with tumor invasiveness, clinical staging, and prognosis.^101^ Ramucirumab, a recombinant human monoclonal antibody targeting VEGFR2, has been extensively studied in various settings for advanced GC or GEJ adenocarcinoma. In the REGARD trial, ramucirumab was compared to placebo in second-line treatment for metastatic GC.^114^ Median overall survival was improved in comparison to placebo, with higher rates of hypertension observed with ramucirumab. Ramucirumab was combined with paclitaxel in the RAINBOW trial, showing a superior progression-free survival rate, but also higher rates of grade >3 adverse effects. These results established ramucirumab plus paclitaxel as a new standard for second-line therapy of advanced and metastatic GC.^115^ Combinations of ramucirumab with other chemotherapy regimens such as FOLFOX did not improve oncological outcomes.^115,116^

### Clinical research progress

There are significant efforts to improve treatment for advanced and metastatic GC which can be divided into two main directions: improvement of drugs or antibodies against known targets such as HER2, PD-1, or VEGF, and development of agents against novel targets, notably FGFR2b. In addition, a number of preclinical studies revealed potential targets, including RNA-binding proteins such as PUM1 or the extracellular matrix receptor DDR1, for which therapeutic agents need to be developed.^117,118^

Given the clinical success of HER2 targeting strategies in metastatic GC, ongoing trials are evaluating modified anti-HER2 antibodies. Margetuximab is derived from trastuzumab with enhanced HER2 binding due to engineered IgG1 Fc domain substitutions^119^, and showed promising results in a phase I study involving 60 patients with HER2-positive solid tumors, including GC.^111^ Results of this trial demonstrated a high rate of cases with stable disease, with mild to moderate adverse events. Margetuximab was then tested in the single-arm, phase Ib-II P-MGAH22-05 trial that enrolled patients with locally advanced or metastatic HER2-positive GC that progressed after at least one prior treatment including trastuzumab plus chemotherapy.^120^ Results of this trial showed objective responses in 17 of 92 evaluable patients. Ongoing research includes a phase II study evaluating margetuximab in combination with immune checkpoint inhibitors, with or without chemotherapy, as first-line treatment for HER2-positive GC (NCT04082364). RC48 is a novel humanized anti-HER2 antibody conjugated to monomethyl auristatin E. RC48 was tested together with toripalimab, an anti-PD1 antibody, in patients with advanced GC or GEJ adenocarcinoma and positive HER2 expression that were refractory to at least one line of treatment.^121^ In patients with GC or GEJ adenocarcinoma, the confirmed objective response rate was 43%. Median progression-free survival was 6.2 months and median overall survival was 16.8 months.

Novel PD-1 targeting antibodies with different pharmacokinetic properties are currently tested in GC including sintilimab and tislelizumab. The ORIENT-16 trial randomized 650 patients in China with unresectable locally advanced or metastatic GC or GEJ adenocarcinoma to receive sintilimab or placebo combined with XELOX chemotherapy.^122^ Sintilimab improved overall survival compared with placebo, particularly in tumors with a CPS score of 5 or higher. The RATIONALE-305 trial across Asia, Europe, and North America for patients with HER2 negative, locally advanced unresectable or metastatic GC or GEJ adenocarcinoma showed that tislelizumab plus chemotherapy significantly improved overall survival compared to placebo plus chemotherapy in all patients, and particularly those with high PD-L1 expression.^112^ Other novel immune targets in GC include VISTA, and antibodies against VISTA are currently tested in early-phase trials, with results still pending (NCT05082610).^123^ In addition, preclinical studies indicate that additional molecular mechanisms and targets for immunomodulation and -evasion exist for GC, such as circularRNAs or RNA-binding proteins.^124,125^

FGFR2 is a novel and promising target as it is highly expressed in subgroups of GC. In the FIGHT study, the efficacy and safety of humanized IgG1 anti-FGFR2b monoclonal antibody bemarituzumab with modified FOLFOX6 was assessed in patients with GC or GEJ adenocarcinoma with high FGFR2b expression.^126^ Median progression-free survival was higher in the bemarituzumab group compared to placebo, with cornea disorder being a specific adverse effect of the experimental drug. Based on these results, bemarituzumab, in combination with chemotherapy, is currently tested in the phase III FORTITUDE-101 trial.

An alternative approach to targeting VEGF with the antibody ramucirumab involves TKIs that block VEGF receptor function. Several such TKIs have been tested in GC or GEJ adenocarcinoma. A notable example is apatinib, a TKI targeting VEGFR2, which underwent investigation in a phase III trial in China.^127^ Patients were randomized to receive apatinib or placebo experiencing progression after two or more lines of chemotherapy, with a median overall survival of 6.5 versus 4.7 months compared to placebo. Based on these results, apatinib was approved in China. Fruquitinib is another small molecule inhibitor targeting VEGFR1-3. In the multicenter phase III FRUTIGA trial, patients with advanced GC or GEJ adenocarcinoma who had progressed on fluorouracil- and platinum-containing chemotherapy were randomized to receive either fruquintinib or placebo, in addition to paclitaxel.^128^ The results were positive for progression-free survival, but not overall survival. Studies for patients outside of Asia are not yet available, and the clinical significance of VEGFR targeting small molecules requires further investigation.

The hepatocyte growth factor receptor MET has pleiotropic effects on proliferation, survival, motility, and angiogenesis, but also drives EMT and tumor invasion.^129^ Studies on MET-positive GCs report a prevalence ranging from 3.8 to 85%, with variability due to different antibodies and scoring systems.^130^ Various strategies targeting MET were tested, however with minor success. The anti-HGF antibody Rilotumumab did not improve survival when added to chemotherapy for untreated MET-positive gastric or GEJ adenocarcinoma in the phase III RILOMET-1 trial.^131^ Similarly, onartuzumab, another fully humanized monoclonal antibody binding to the MET receptor’s extracellular domain, did not enhance overall survival when combined with chemotherapy in untreated MET-positive GEJ adenocarcinomas, as reported in the phase III MET-Gastric trial.^132^ Tivantinib is a small molecule inhibitor of c-MET and was evaluated in a phase II trial including patients with advanced GEJ cancer receiving standard FOLFOX and tivantinib. Median progression-free survival was 6.1 months and overall survival was 9.6 months, which were consistent with historical controls for first-line FOLFOX therapy.^120^

In summary, with the emergence of novel targets such as CLDN18.1 and FGFR2b as well as improved HER2 and immune checkpoint targeting antibodies, the major challenge will be to optimize the molecular stratification of GC and to identify synergistic combinations of targeted agents.

## Biliary tract cancer

### Epidemiology

Biliary tract cancer or cholangiocellular carcinoma (CCA) comprises a heterogenous group of malignancies that can arise from the intrahepatic (iCCA), the perihilar (pCCA), or the extrahepatic biliary tree distant from the cystic duct (dCCA). Extrahepatic CCA accounts for up to 90% of CCA (50–60% pCCA and 20–30% dCCA respectively).^121^ All CCA accounts for 15% of all primary liver tumors and 3% of digestive cancers.^122^ The incidence and mortality of CCA varies internationally. In European and North American countries the annual incidence is reported to be 0.35–3.59 cases per 100,000 population, whereas it is significantly higher in South America and the Asia-Pacific region (1.12–12.42 cases per 100,000 population).^133–135^ Annual mortality was reported with 173.974 deaths globally in 2017.^136^ Despite the fact that CCA is a rare disease, its incidence and mortality is rising globally, primarily due to an increase in iCCA.^122,137^ From 1990–2017 the global incidence increased by 76% and the global number of deaths increased by 65%, notably with a high regional variability.^136^ These epidemiologic changes are also partly caused by changes in ICD classification and substantial misclassification in the past.^126,138–140^ Furthermore, improved diagnostic tools resulted in a higher detection rate and a more accurate distinction between cancer of unknown primary and iCCA. Detection of combined hepatocellular carcinoma/iCCA due to higher rates of biopsies might be another reason for this increase.^141^ The global variation in incidence and mortality is possibly due to differential risk factors and other genetic predispositions.^126,142^ While the leading cause of CCA is a liver fluke in Southeast Asia, etiology in the Western world remains unknown in more than half of the cases. All the known risk factors result in chronic biliary inflammation and bile stasis.^142^ The strongest risk factors that are shared by iCCA and p/dCCA are cysts and stones in the bile ducts, cirrhosis, and infection with hepatitis B and C virus. A rising risk factor is diabetes.^140,142^ Furthermore, high alcohol consumption and tobacco smoking are associated with CCA development.^140,142^ Patients presenting with CCA are typically between 50 and 70 years, but in cases of concomitant diseases such as primary sclerosing cholangitis or choledochal cysts, patients can present at a younger age.^141–144^ The overall incidence of CCA is higher in males than females, but the underlying reasons are unclear.^143,145^

### Molecular pathology and tumor biology

The molecular landscape of CCA is heterogeneous, reflecting the different etiologies and locations of the disease. As up to 50% of CCA harbor potentially actionable genetic alterations, hence a profound understanding of tumor biology and the distinct molecular subtypes is essential to identify tailored therapies.^146^ Carcinogenesis of CCA is a complex process involving genomic and epigenetic alterations that regulate processes such as DNA repair, receptor tyrosine kinase signaling, and epigenetic regulation.^129^ Pre-invasive lesions can precede all subtypes of CCA.^147,148^ pCCA and dCCA mainly present as mucin-producing adenocarcinomas or papillary tumors.^149^ Rare histological variants include intestinal-type CCA, mixed HCC-CCA, and lymphoepithelioma-like CCA.^148,150,151^ In iCCA a controversy exists over the definite origin of cancer cells.^152^ Small bile duct iCCA are thought to originate from hepatic stem or progenitor cells and are mostly non-mucin producing. They characteristically harbor IDH mutations and FGFR2 fusions.^149^ Small bile duct iCCA frequently develops in chronic liver diseases like viral hepatitis or cirrhosis that is known to induce the activation of hepatic progenitor cells.^153–155^ This is in line with findings in subtypes of HCC and mixed HCC-CCA, where hepatic progenitor cells are also involved.^154^ Large bile duct iCCA are mostly mucin-producing tumors that derive from mucous cholangiocytes or peribiliary glands, possibly from precancerous lesions.^153,156^ In these subtypes, chronic inflammation as in PSC or liver-fluke infection is most relevant.^156,157^ Mutational patterns vary depending on the anatomic location of CCA.^128^ While *FGFR2* fusions are almost exclusively found in iCCA, *PRKCA–PRKCB* fusions are more characteristic of dCCA and pCCA.^146,158,159^ In fluke-related CCA, chronic inflammation might lead to epigenetic changes and subsequently tumor development.^160,161^ In these tumors, mutations in *TP53*, *ARID1A*, *ARID2*, *BRCA1*, and *BRCA2*, as well as non-coding mutations in promoters associated with H3K7me3, are more prevalent.^162^ Furthermore, *ERBB2* amplifications and a hypermethylation phenotype can be frequently observed.^162^ There are two fluke-related subtypes. One that harbors mutations in genes like ECT2 leading to checkpoint defects (C1) and another with altered bile acid metabolism, T cell infiltration, which is linked to obesity (C2).^159^ In non-fluke-related CCA, genetic driver mutations occur that lead to epigenetic changes.^160,161^ In this group, inactivating mutations in *PBRM1, BAP1, PIK3CA*, and *ELF3*, and gain-of-function mutations in *IDH1* and *IDH2* are more prevalent.^146,161–164^ Additionally, *FGFR* translocations, enrichment of *PRKACA* and *PRKACB*, and hypermethylation in promoter CpG shores are predominantly found.^144,146,162,165,166^ CCA are highly desmoplastic cancers with a rich tumor immune microenvironment.^167^ The tumor microenvironment of CCA is immunosuppressive, characterized by an abundance of suppressor T-cells that overexpress co-inhibitory receptors such as PD-1, and decreased amount of cytotoxic immune cells.^168^

The mutational landscape of CCA is gradually unraveled by tumor genome sequencing studies.^18,144,161,162,165,169^ Pathways that are genetically altered in CCA include WNT, MYC, ERBB, TNF, and VEGF signaling.^170^ In general, pCCA/dCCA have high frequencies of *KRAS* and *TP53* mutations.^146,171,172^ The aim of molecular profiling is to define subtypes of CCA that predict response to specific therapies and oncological outcomes.^170,173^ For example, RAS/BRAF mutational status can predict overall survival in patients with iCCA.^170^ Other profiling studies grouped patients with iCCA into an inflammation or proliferation type.^173^ Different pathways are activated in these two subtypes. For example, overexpression of cytokines and activation of STAT is found in the inflammation type while KRAS/BRAF mutations are more frequent in the proliferation type and associated with worse clinical outcomes.^173^ With regard to pCCA and dCCA, metabolic, proliferation, mesenchymal, and immune subtypes have been described.^173^ As the mutational landscape of CCA is further elucidated, several targeted therapeutic options have arisen of which many have been implemented into current therapeutic strategies.

### Current management

**Fig. 3:**
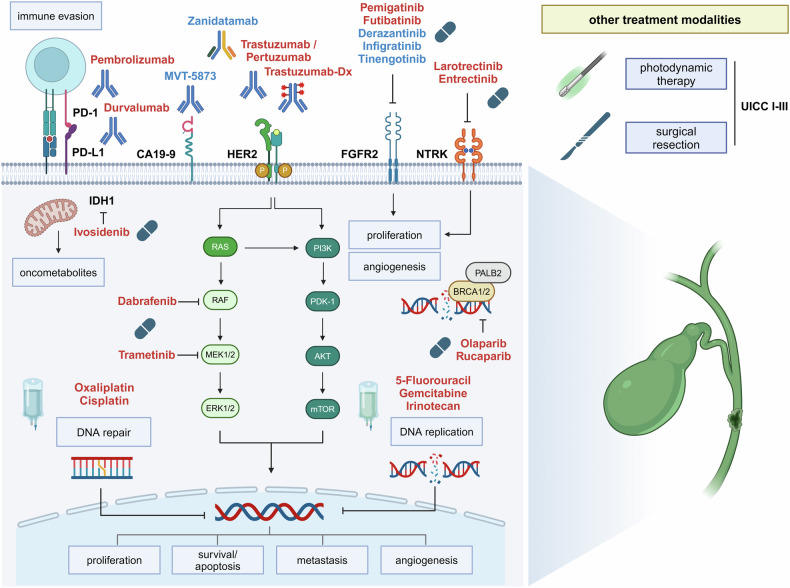
Current therapeutic targets in biliary tract cancer. 5-FU 5-fluorouracil, FGFR2 fibroblast growth factor receptor 2, HER2 human epidermal growth factor receptor 2, PD-1 programmed cell death protein 1, PD-L1 programmed cell death ligand 1, PD-L1 programmed cell death 1 ligand 1, NTRK neurotrophic tyrosine receptor kinase, CA19-9 carbohydrate antigen 19-9. Therapies that are recommended by international guidelines are marked in red. Therapies with preclinical or only low levels of clinical evidence are highlighted in blue

CCA has an overall poor prognosis despite recent progress in diagnostic methods and therapies. Over the last decade, the treatment of CCA has fundamentally changed as tumor biology is better understood and new targeted therapeutic strategies are introduced to the management of metastatic CAA (see Fig. 3).^174–178^

### Management of resectable biliary tract cancer

#### Adjuvant chemotherapy

Surgery remains the main curative treatment for CCA, but tumor recurrence is observed in more than half of patients who underwent surgical resection, leading to 5-year survival rates that do not exceed 7–20%. Since CCA is a disease with high rates of relapse, adjuvant treatment strategies are highly relevant.^179,180^ Currently, adjuvant therapy is recommended for all patients based on the results of the BILCAP trial.^181^ In this randomized, multicenter phase III study, 447 patients with resected CCA and gallbladder cancer were randomized to receive either capecitabine or observation.^182^ Although the primary endpoint, overall survival, was not met in the intention-to-treat analysis, capecitabine was suggested to improve survival in the per-protocol analysis. This led to the recommendation of 6 months of capecitabine treatment in patients with resected CCA.^181^ Two other phase III randomized clinical trials evaluating gemcitabine (BCAT trial) or gemcitabine and oxaliplatin (PRODIGE-12 trial) showed no benefit of these regimens in an adjuvant setting.^183,184^ The ESPAC-3 phase III trial observed a survival benefit of either 5-fluorouracil or gemcitabine as adjuvant therapy when compared to placebo after adjusting for prognostic variables.^185^ The phase III JCOG1202 ASCOT trial investigated adjuvant therapy with S-1 versus observation alone, and could show a significant survival benefit.^186^ This led to the recommendation of S-1 as standard adjuvant therapy in Japan. There are several ongoing trials investigating different adjuvant therapeutic regimens such as gemcitabine plus cisplatin versus capecitabine (ACTICCA-1)^187^ in Europe or gemcitabine plus cisplatin versus gemcitabine plus S-1 (KHBO1901) in Asia.

### Management of unresectable or metastatic biliary tract cancer

Only 20–30% of CCA are considered resectable at diagnosis^181,188,189^, hence most patients require pharmacological therapy. The treatment of advanced or metastatic CCA has significantly changed over the last years. While chemotherapy remains the main therapeutic backbone for most patients with CCA, immunotherapy has become an integral part of first-line therapy. Furthermore, an expanding spectrum of targeted therapies has become available for CCA with specific driver mutations, highlighting the importance of early molecular diagnostics to optimize therapy. In clinical practice, we recommend to perform panel or exome sequencing of tumor biopsies at initial diagnosis to determine potentially actionable gene mutations and fusions (including BRAF, FGFR, IDH1, IDH2, BRCA1, BRCA2, and NTRK) for further lines of therapy. Furthermore, MMR/MSI status and expression of HER2 should be routinely assessed by immunohistochemistry.

#### Chemotherapy and immune checkpoint inhibitors

In patients with unresectable or metastatic CCA, chemotherapy can provide a survival benefit.^190^ In 2010, the ABC-02 trial established gemcitabine and cisplatin as standard first-line chemotherapy.^191^ Since 2022, gemcitabine and cisplatin plus immune checkpoint inhibitors (pembrolizumab/durvalumab) has become the new standard first-line treatment for unresectable CCA. The addition of immune checkpoint inhibitors was introduced based on the results of two randomized trials. In the TOPAZ-1 trial, 685 treatment-naive patients with advanced CCA received gemcitabine and cisplatin with either durvalumab or placebo^192^, followed by maintenance therapy with single-agent durvalumab in the treatment group. Overall survival was significantly increased in the durvalumab versus placebo group (12.8 versus 11.5 months), with comparable toxicity profiles.^192^ In the KEYNOTE-966 trial, 1069 treatment-naive patients with advanced CCA received gemcitabine and cisplatin either with pembrolizumab or placebo for up to 8 cycles.^193^ Subsequently maintenance therapy with gemcitabine plus immunotherapy or placebo was possible. A survival benefit was shown in the immunotherapy group (12.7 months versus 10.9 months). More extensive chemotherapy regimens such as FOLFIRINOX (PRODIGE 38 AMEBICA) or gemcitabine and cisplatin plus nab-paclitaxel (SWOG 1815) failed to improve overall survival in first-line therapy or had unfavorable toxicity profiles.^194,195^ A meta-analysis showed a modest benefit of second-line chemotherapy in advanced or metastatic CCA.^196^ Based on the results of the ABC-06 trial, FOLFOX is a standard regimen in second-line therapy for CCA, as it improved median survival (6.2 versus 5.3 months) after progression on gemcitabine and cisplatin.^197^ Alternative regimens include monotherapies with 5-fluorouracil, capecitabine or irinotecan or combination of capecitabine with oxaliplatin or irinotecan.^198^ The role of irinotecan in second-line chemotherapy remains controversial. A phase II clinical trial conducted in China showed survival benefits of irinotecan containing regimens in second-line therapy, as well as the Phase IIb NIFTY trial conducted in South Korea.^199^ However, the European Naliricc phase II trial could not observe a survival benefit when liposomal irinotecan was added to 5-fluorouracil.^200^

#### Advanced or metastatic FGFR2 fusion-positive CCA

Fibroblast growth factor receptor (FGFR) signaling is involved in the regulation of cell proliferation, survival, migration, and differentiation and is deregulated in many cancers.^201,202^ FGFR2 alterations can be found in 10–15% of CCA, leading to activation of different signaling pathways.^163,169,203–206^ The FIGHT-202 trial, a single-arm phase II study, investigated the use of pemigatinib, an oral FGFR1-3 inhibitor in patients with advanced or metastatic CCA with or without FGFR alterations. Objective response rate in patients with FGFR2 fusions or rearrangements was 35.5%.^203^ Most common grade 3 or 4 toxicity was hyperphosphatemia that occurred in 12% of patients.^203^ In the phase II FOENIX-CCA2 trial, treatment with futibatinib, a selective, irreversible FGFR1-4 inhibitor, achieved an overall response rate (ORR) of 42% in patients with FGFR2 fusions. Both agents have been approved by the FDA. Comparable results have been published for derazantinib, a FGFR1-3 inhibitor, with a phase II study showing an ORR of 21.4%.^207^ Other FGFR inhibitors are subject to ongoing trials, comparing FGFR inhibitors to standard cytotoxic chemotherapy or aiming to overcome chemoresistance after initial FGFR inhibitor treatment. Pemigatinib (FIGHT-302)^208^, infigratinib (PROOF-301)^209^, and futibatinib (FOENIX-CCA3)^210^ are investigated in ongoing phase III trials, comparing FGFR inhibition to standard-of-care first-line treatment with gemcitabine and cisplatin (without immune checkpoint inhibitors).^193,194^ Assessment of combination therapies is also ongoing. Derazantinib plus atezolizumab is investigated in the phase II ADVANCE trial as second-line therapy in patients with FGFR2 gene alterations (NCT05174650). Acquired resistance against FGFR inhibitors by secondary mutations in the kinase domain of FGFR2 is a common phenomenon.^211,212^ The FGFR2-selective inhibitor lirafugratinib might overcome this resistance and avoid the toxicity of pan-FGFR inhibitors. In a phase I/II trial that included 91 patients with CCA, 74 patients showed partial response (64%) with an overall favorable toxicity profile.^213^

#### Advanced or metastatic IDH1 and IDH2 mutated CCA

Mutations in *IDH1* and *IDH2* genes can lead to oncogenic effects by DNA hypermethylation and impairing cell differentiation.^214^ Up to 15% of patients with iCCA harbor IDH1 mutations with a large variety across geographic regions.^172,215^ In pCCA and dCCA these mutations are less frequent (0.8%).^215^ The oral IDH1 inhibitor ivosidenib was compared to placebo in pretreated CCA patients in the phase III ClarIDHy trial. Significant effects on progression-free survival were observed (2.7 versus 1.4 months). Overall survival was prolonged in the ivosidenib group with 10.3 versus 7.5 months, but this did not reach statistical significance due to the crossover design.^216^ Based on these results, ivosidenib was approved by the FDA in 2022. However, resistance to ivosidenib develops in all patients demanding for sequential therapies.^217^ Several trials are on the way to investigate other IDH1 inhibitors such as olutasidenib (FT-2102, NCT03684811), LY3410738 (NCT04521686), and HMPL-306 (NCT04762602) that might be able to bind even when resistance to ivosidenib has developed.^217^ *IDH2* mutations can occur as primary or secondary resistance mechanisms.^218,219^ Enasidenib, an IDH2 inhibitor, is FDA approved for *IDH2* mutant AML and combination therapy with ivosidenib might be a rationale to overcome resistance in patients with CCA.^220^ Furthermore, *IDH1/2* mutations have been described to sensitize tumors to PARP inhibition as they suppress homologous recombination.^221^ Given this rationale, olaparib is investigated in an ongoing phase II study in IDH1/2 mutant patients with solid tumors (NCT03212274).

#### Advanced or metastatic BRAF V600E mutated CCA

BRAF mutations occur in up to 5% of CCA, with BRAF V600E being the most common alteration.^222^ Vemurafenib was investigated in a phase II basket trial in patients with BRAF V600E mutated tumors, and one partial response was observed in eight patients with CCA.^223^ Combination of MEK- (trametinib) and BRAF-inhibition (dabrafenib) was effective in a phase II basket trial (ROAR) in 43 CCA patients with a reported objective response of 51% and a median overall survival of 14 months^224^, leading to its approval in 2022.

#### Advanced or metastatic HER2 mutant/amplified CCA

HER2 is an oncoprotein belonging to the ErbB family and a well-known actionable target in precision oncology. Overexpression of HER2 protein or amplification of the HER2 gene occurs in up to 20% of CCA patients and is more common in patients with pCCA and dCCA (17.4%) than in iCCA (4.8%).^225–227^ Dual HER2 blockade with trastuzumab and pertuzumab was evaluated in 39 patients with CCA in the phase IIa MyPathway basket study. The objective response rate was 23% and the median progression-free survival was 4 months. As part of a basket phase II trial (SGNTUC-019) 29 patients with metastatic CCA and HER2 overexpression received a dual HER2 blockade with tucatinib and trastuzumab. ORR in this cohort was 47% with a median progression-free survival of 5.5 months.^228^ The antibody-drug conjugate trastuzumab-deruxtecan showed an ORR of 36.4% in 22 HER2-positive patients with CCA, including two complete responses, in the HERB phase II trial.^229^ Trastuzumab-deruxtecan was further assessed in 267 patients with metastatic CCA in the phase II DESTINY-PanTumor02 trial.^230^ Forty-one patients with CCA were included and showed an ORR of 22.0%. The response was more pronounced in patients with high expression levels of HER2. Zanidatamab, a bispecific antibody that binds two distinct HER2 epitopes showed an ORR of 41% and a median progression-free survival of 5.5 months in 80 patients with HER2-positive CCA.^231^

#### Advanced or metastatic CCA with NTRK and RET fusions

Fusions of *NTRK1, NTRK2*, and *NTRK3* genes are rare alterations in CCA (0.7–1.8% of cases).^232,233^ Two basket trials investigated larotrectinib and entrectinib in TRK-fusion-positive cancers involving three patients with CCA. Of those, two patients with CCA had a partial response and one disease progression. Both larotrectinib and entrectinib are approved in TRK-fusion-positive solid tumors. RET fusions are also a rare condition in CCA (up to 0.15%).^234^ In the phase I/II ARROW trial, pralsetinib, a selective inhibitor of RET, was evaluated in RET fusion-positive patients. ORR was 57% (95% CI, 35–77) among all patients.^235^ Three patients with CCA were included, of whom two had partial responses and one stable disease.^235^ Pralsetinib is further investigated in the phase II TAPISTRY trial (NCT04589845). Selpercatinib, another RET selective receptor tyrosine kinase inhibitor was investigated in the phase I/II basket trial (LIBRETTO-001).^236^ Of the 41 patients, ORR was 43.9% (95% CI 28.5–60.3), two patients had CCA and achieved partial responses.^236^

### Clinical research progress

Currently, major strategies to optimize the management of CCA include improving therapeutic agents for the treatment of tumors with specific molecular alterations (as outlined in the section above) and identifying effective drug combinations. Another important challenge is the detection of drug resistance and the subsequent adaptation of therapy.

Many chemotherapies and targeted therapies have been evaluated in combination with immune checkpoint inhibitors in CCA, with mixed results. The combination of tyrosine kinase inhibitor lenvatinib and pembrolizumab was investigated in patients with pretreated CCA in the LEAP-005 phase II trial, and ORR was 10% in this cohort.^237^ Another single-arm study investigated this combination in 32 pretreated patients with CCA. Here, median progression-free survival was 4.9 months with an ORR of 25% and DCR of 78.1%.^238^ Combination of nivolumab with the CTLA-4 inhibitor ipilimumab was assessed in a cohort from the CA209-538 trial, a phase II open-label basket study.^239^ In 39 patients with pretreated CCA (10 p/dCCA, 16 iCCA) ORR was 23% with a disease control rate of 44%. None of the included tumors were dMMR/MSI. Another phase II trial (BilT-01) compared the combination of gemcitabine/cisplatin with nivolumab versus CTLA-4 inhibitor ipilimumab plus nivolumab as first-line therapy.^240^ Median progression-free survival was 6.6 months in the chemotherapy plus nivolumab arm versus 3.9 in the ipilimumab/nivolumab arm. Addition of durvalumab and CTLA-4 inhibitor tremelimumab to gemcitabine/cisplatin in first-line therapy in CCA patients was investigated in a single-center phase II study.^241^ Group A, comprising 32 patients received chemotherapy followed by chemotherapy plus durvalumab and tremelimumab. Group B (49 patients) received chemotherapy plus durvalumab from the beginning, and group C (47 patients) received chemotherapy plus durvalumab and tremelimumab from the beginning. ORR was 50% in cohort A, 72% in cohort B, and 70% in cohort C, and the addition of dual immunotherapy did not improve therapeutic outcomes.^241^ There are several ongoing trials investigating combinations of immunotherapy with other therapeutic agents in CCA such as chemotherapy plus combined VEGF and PD-1 blockade, that showed promising results with ORR of 80% in a phase II trial.^242^ Combination of immunotherapy with targeted strategies is also the subject of several trials. The MEK inhibitor cobimetinib added to atezolizumab was investigated in a phase II trial and showed a progression-free survival of 3.6 months in the combination arm versus 1.9 months in the monotherapy arm.^243^ Another phase II trial investigating bintrafusp alfa, a bifunctional fusion protein did not meet its primary endpoint with ORR of 10.7% targeting PD-L1 and TGF-β in patients with pretreated CCA.^244^ Combination of chemotherapy with immunotherapy has become standard of care in the first-line therapy of CCA. However, the role of combined immunotherapies or combination of immunotherapy with other agents remains so far without significant success.

In conclusion, CCA is a highly heterogeneous group of tumors due to different etiologies and molecular characteristics. To therapeutically address this heterogeneity, precision-medicine based strategies are required and enrollment in distinct, genomics-driven clinical trials would be favorable. Targeted strategies, such as FGFR2 or IDH1/2 inhibitors have already been approved and are part of standard management of advanced or metastatic CCA. However, a better understanding of underlying mechanisms in carcinogenesis and cancer progression is necessary to enlarge the patient group that can benefit from a tailored therapy approach.

## Hepatocellular cancer

### Epidemiology

Hepatocellular carcinoma (HCC) is the most common primary liver cancer and presents a significant global health challenge, ranking as the sixth most common cancer and responsible for ~758,000 deaths worldwide in 2022, according to GLOBOCAN data.^2^ The incidence of HCC is intricately linked to liver cirrhosis, with ~3–4% of patients with liver cirrhosis progressing to HCC annually.^245^ Traditionally, regions with high rates of chronic hepatitis B virus (HBV) and hepatitis C virus (HCV) infections, such as East Asia and Sub-Saharan Africa, have reported the highest incidences of HCC.^246,247^ Latest projections predict a continuously increasing global incidence of HCC solely on the basis of viral hepatitis.^248^ In addition, metabolic dysfunction-associated steatotic liver disease has emerged as an even more prevalent driver of HCC.^249^ With a rapidly increasing global obesity epidemic and the associated metabolic syndrome, the epidemiology of HCC is evolving even more.^250^

### Molecular pathology and tumor biology

HCC is a complex, multifactorial disease with diverse etiology mostly leading to liver cirrhosis and subsequently to cancer development. The most significant risk factors are chronic viral hepatitis infections, specifically HBV and HCV, which lead to chronic inflammation and liver cell damage, promoting genetic mutations and aberrant cell growth.^231^ Alcohol abuse, causing alcoholic liver disease and cirrhosis, and MASLD, driven by the global obesity epidemic, also increase HCC risk.^249,251^ Exposure to aflatoxin B1, a potent hepatocarcinogen, poses a major risk, especially in regions with high dietary aflatoxin levels such as (South-)East Asia and Middle and Western Africa.^252,253^ Genetic predisposition, iron overload in hereditary hemochromatosis, and α-1 antitrypsin deficiency are additional factors associated with increased HCC susceptibility.^254^ The pathogenesis of HCC involves chronic inflammation, creating a microenvironment that favors tumor growth and immune evasion.^236^ HBV and HCV infections contribute to this inflammation through viral replication, immune cell activation, and pro-inflammatory cytokine production.^255^ These signals activate pathways and key oncological drivers such as TGF-β, Wnt/β-catenin, Notch, EGF, HGF, VEGF, SHH, and YAP/TAZ, among others, ultimately enhancing cell survival, proliferation, and evasion of apoptosis.^256^ Epigenetic changes, including DNA methylation and histone modifications, silence tumor suppressor genes and activate oncogenes.^257^ Altered microRNA expression further disrupts post-transcriptional regulation, impacting various cellular processes.^258^ However, in contrast to CCA, these targets have not yet resulted in the development of novel therapeutic strategies in HCC.

### Current management

#### Prevention

The well-defined at-risk population for HCC allows for regular surveillance, facilitating early detection. A European meta-analysis including 59 studies and 145,396 patients showed that six-monthly surveillance using ultrasound significantly improves early tumor detection, increasing eligibility for curative therapy and extending patient survival.^259^ Vaccination effectively prevents HCC arising from hepatitis B.^260^ Testing for HBV and HCV is crucial, and treating these infections with nucleos(t)ide analogs (NUCs) or direct-acting antivirals (DAA) can reduce cirrhosis and HCC risk. In addition, NUCs therapy for HBV may prevent HCC recurrence, though this effect is not clearly proven for HCV therapy.^261^ Just recently several studies reported that HCC patients achieving sustained virological response (SVR) under DAA treatment for HCV still require indefinite surveillance due to persistent recurrence risk.^245^ Finally, recent studies suggest potential preventive effects of medications like statins, metformin, and acetylsalicylic acid (ASA).^262^ There is also speculation about an inverse correlation between coffee consumption and a 40% reduction in HCC risk.^263^

#### Barcelona clinic of liver cancer classification (BCLC)

The BCLC staging system classifies patients into five stages based on tumor size, number, and location, considering patients’ performance status and liver function.^264^ Early-stage patients with good performance status and preserved liver function may undergo liver transplantation, resection, or local ablation, with an excellent prognosis. Intermediate-stage patients are typically treated with TACE, while advanced-stage patients receive systemic treatment.^265,266^ The advent of immunotherapies has sparked debates on offering systemic treatment to intermediate-stage patients, with ongoing clinical trials exploring this approach.

### Management of resectable disease

#### Early-stage HCC

Surgical approaches, including liver resection and liver transplantation, are primary curative treatments for early-stage HCC.^265,267,268^ Advances in surgical techniques and perioperative care have improved outcomes, particularly with minimally invasive techniques like robotic-assisted or laparoscopic surgery. Resection of tumors ≤2 cm was shown to result in a 5-year survival rate of 63–70%. However, surgery (and local ablation) of HCCs in cirrhotic livers has a high recurrence or de novo rate of 60–70%.^269^ Of note though, surgery in patients with liver cirrhosis remains challenging due to increased mortality. Retrospective analyses from the Veterans Affairs Surgical Quality Improvement Program reported a six times higher postoperative mortality in patients with liver cirrhosis compared to patients with elective surgery.^270^ The Milan criteria (single nodule ≤5 cm, up to three nodules ≤3) define eligibility for liver transplantation, with efforts to expand criteria and explore downstaging approaches showing promising results.^271,272^ Despite high recurrence rates after resection, liver transplantation offers a significant survival advantage by removing both the tumor and the cirrhotic liver, reducing the risk of recurrence and new tumor formation. Data from the European Liver Transplant Registry showed a 70% 5-year survival of patients with tumors within Milan criteria who received liver transplantation.^273^ Locoregional therapies, such as radiofrequency ablation (RFA) and microwave ablation (MWA), provide valuable alternatives to surgical resection for small, unresectable tumors. RFA studies reported a 5-year survival rate between 40 to 70%.^274^ These minimally invasive approaches preserve surrounding healthy liver parenchyma and are well-suited for patients with small tumors <3 cm.^274^ External beam radiation may be considered in selected patients, with retrospective data suggesting non-inferiority to RFA, although larger prospective trials are needed.^275^

### Management of unresectable or metastatic HCC

#### Intermediate stage

TACE involves injecting chemotherapeutic agents into the hepatic artery supplying the tumor, followed by embolization to induce ischemic tumor necrosis. It is considered the treatment of choice for intermediate-stage HCC with well-preserved liver function. For nearly a decade the theoretical advantage of combining TACE with systemic targeted therapies was unsuccessfully evaluated. The rationale for combining TACE with TKIs was that TACE could reduce tumor burden locally, while TKIs could target systemic disease and reduce angiogenic responses induced by TACE. However, multiple phase II and III studies evaluating a combination with sorafenib (sorafenib SPACE trial^276^, TACE2 trial^277^, and TACTICS trial^278^), Orantinib (Oriental^279^), or adjuvant brivanib (BRISK-TA trial^280^), have failed to demonstrate a significant increase in efficacy compared to monotherapies. Finally, real-world data on TACE combined with camrelizumab and apatinib for patients did not show any benefit.^255^ However, combination therapy may become of interest utilizing novel immune therapeutics such as durvalumab. Just recently the EMERALD-1 study, a phase III clinical trial assessing the efficacy and safety of TACE with durvalumab and bevacizumab in patients with unresectable HCC demonstrated improved progression-free survival with combination treatments.^281^ Of note, selective internal radiotherapy (SIRT) with Yttrium-90 (90Y) resin microspheres had been proposed in patients with intermediate HCC with locally advanced or intermediate-stage HCC after unsuccessful TACE. However, the phase III SARAH trial did not show a significant difference in overall survival between the two groups.^282^

#### Advanced stage, systemic therapy

**Fig. 4:**
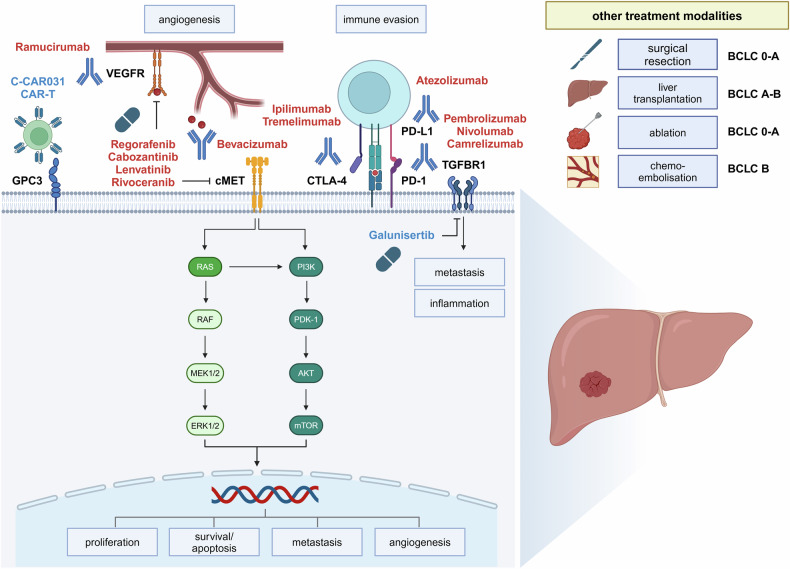
Current therapeutic targets in hepatocellular cancer. VEGFR vascular endothelial growth factor receptor, CTLA-4 cytotoxic T-lymphocyte-associated protein 4, PD-1 programmed cell death protein 1, PD-L1 programmed cell death ligand 1, PD-L1 programmed cell death 1 ligand 1, TGFBR1 transforming growth factor beta receptor 1, GPC3 glypican-3, BCLC Barcelona clinic of liver cancer classification. Therapies that are recommended by international guidelines are marked in red. Therapies with preclinical or only low levels of clinical evidence are highlighted in blue

Systemic targeted therapies have revolutionized the treatment of advanced HCC (see Fig. 4). Sorafenib, the first FDA-approved targeted therapy (SHARP Trial, Asia-Pacific Trial)^283^, paved the way for other TKIs such as lenvatinib for first-line treatment (REFLECT trial)^284^, regorafenib (RESORCE trial)^285^, cabozantinib (CELESTIAL trial)^286^ and the anti-VEGF antibody ramucirumab (REACH-2 trial).^287^ However, immune checkpoint inhibitors have opened a completely new chapter in HCC systemic therapy with significantly improved overall survival rates emerging as a promising approach. Atezolizumab (PD-L1 inhibitor) and bevacizumab (anti-VEGF antibody) have shown significant efficacy in combination for HCC. The IMbrave150 trial established this combination as a first-line treatment for unresectable HCC.^15^ In addition, the combination of single tremelimumab and continued durvalumab (STRIDE regimen) showed significant improvement in overall survival compared to sorafenib.^288^ Lately, camrelizumab plus rivoceranib also showed significant improvements in overall survival and progression-free survival compared to sorafenib.^289^ However, another combination of immune checkpoint inhibitor and TKI, did not show a survival benefit. In the phase 3 LEAP-002 trial which combined pembrolizumab with the tyrosine kinase inhibitor lenvatinib, the combination therapy did not improve progression-free or overall survival compared to lenvatinib monotherapy.^182^ In light of multiple available therapeutic strategies, subgroup analyses of the atezolizumab plus bevacizumab and STRIDE regimens opened discussions about efficacy of atezolizumab plus bevacizumab in metabolic derived HCC or STRIDE in HCV patients.^287,288^ Furthermore, recent data on the development of immune-related adverse events (irAEs) during treatment was demonstrated to be associated with better clinical outcomes, suggesting that these events could serve as indicators of a positive therapeutic response. In turn, effective and stringent management of irAEs effectively allows patients to continue benefiting from these potent immunotherapies. Subsequent treatment after progression on these regimens currently remains unclear. Therapeutic options include both tyrosine kinase inhibitors like sorafenib or lenvatinib, but also alternative immunotherapies. These options and therapy sequences will have to be evaluated in future clinical trials. Due to the lack of predictive biomarkers and actionable genetic alterations, molecular pathology is currently not required for the management of unresectable or metastatic HCC.

### Clinical research progress

The research landscape for HCC is advancing in several areas aimed at improving patient outcomes across different stages of the disease. Current key developments include adjuvant immunotherapy, combination of TACE and immunotherapy, comparison of TACE versus immunotherapy, combination immunotherapy, and subsequent therapy lines post-immunotherapy.

Despite successful surgery, the risk of recurrence is high. Adjuvant therapy targets residual microscopic disease to potentially reduce recurrence and extend both disease-free and overall survival, possibly shifting the standard of care. Currently, the IMBrave050 trial is investigating the role of adjuvant immunotherapy with atezolizumab plus bevacizumab following surgical resection and demonstrated a benefit on recurrence-free survival over active surveillance.^290^ However, data on overall survival were still immature and are certainly critical for approval and routine clinical use of atezolizumab and bevacizumab after resection or tumor ablation.^290^ Similar trials are ongoing using durvalumab plus tremelimumab or camrelizumab plus rivoceranib, and also other therapeutic combinations.^291^ The EMERALD-1 phase III clinical trial assessed the efficacy and safety of this combination, showing improved progression-free survival. This study underscores the potential benefits of integrating combination therapies into clinical practice to enhance overall survival rates.^281^ Given the availability of novel and significantly more effective systemic treatment options for HCC, treatment modalities for the intermediate stage are currently being intensively discussed. The currently ongoing ABC-HCC trial investigates if novel immunotherapy combinations may even outperform TACE in earlier stages, particularly the BCLC intermediate stage. The trial is currently recruiting and would—if positive—certainly change the treatment landscape for HCC.^292^ Recently, the CheckMate 9DW trial has demonstrated that using nivolumab and ipilimumab as a first-line treatment significantly improves overall survival in patients with untreated, unresectable HCC compared to TKI treatment and reported a median overall survival of 23.7 months.^293^ Thus, this combination may soon add to the currently available therapeutic arsenal. Finally, with the rapid evolution of novel first and second-line treatment options in HCC, the optional sequence of treatment remains to be investigated. Particularly after the failure of immunotherapy, subsequent treatment options remain widely unexplored. Even more general questions like the feasibility of alternative immunotherapy after initial immunotherapy failure but also the efficacy of available second-line options cabozantinib, regorafenib, ramucirumab after immunotherapy (instead of sorafenib first-line therapy) remain to be urgently investigated. Research is increasingly focusing on second-line therapies following the failure of initial immunotherapy, which is crucial for continuous care in HCC. As resistance to first-line immunotherapies emerges, identifying effective second-line treatments, including novel combinations of therapies like tyrosine kinase inhibitors or other immune modulators, is essential for managing disease progression and improving patient survival. Overall, these efforts underscore a dynamic shift towards more effective but potentially also more personalized management strategies in HCC, aiming not just to extend life but also to improve the quality of life for patients at various disease stages.

## Pancreatic cancer

### Epidemiology

The pancreas originates from different embryological tissue types, which reflect the different functions of the pancreas. Most cells are part of the exocrine pancreas, which is formed by ductal and acinar cells. In contrast, the endocrine pancreas contains endocrine cell types (α, β, δ, and F), which secrete hormones like glucagon, insulin, or somatostatin. Approximately 80–90% of pancreatic malignancies are adenocarcinomas and arise from the ductal cells of the exocrine pancreas.^294^ Therefore, the terms “pancreatic cancer” and “pancreatic ductal adenocarcinoma” are often used synonymously and this review will focus on PDAC. Malignancies arising from the acinar cells are rare, as are malignancies from the endocrine pancreas (pancreatic neuroendocrine neoplasms).^295^ In 2022, approximately 510,566 cases of pancreatic cancer were diagnosed globally, with 467,005 patients who died of the disease.^2^ A steady increase in the incidence of PDAC has already been predicted in the past.^296^ However, according to the latest statistics, the number of PDAC cases appears to be rising even faster than predicted.^296,297^ Increased incidence affects not only the US, but virtually every developed country.^298^ Unfortunately, the prognosis of PDAC remains poor. The 5-year survival rate has only slightly increased to approximately 10%.^299^ The reason for the poor prognosis lies in a long asymptomatic disease interval, aggressive tumor biology, early (micro-) metastasis, and a high resistance to various forms of therapy. Taken together, it is not surprising that PDAC is estimated to be the second leading cause of cancer-related death before 2030, second only to lung cancer. The reasons for the dramatically rising incidence of PDAC is not yet fully understood, but may be partially explained by an increased relevance of exogenous risk factors. The most important exogenous risk factors are cigarette smoking, obesity, and heavy alcohol abuse. In contrast to other smoking-related malignancies, a unique genetic signature for smoking-related PDAC has not yet been identified. In mice, nicotine promotes pancreatic carcinogenesis via downregulation of Gata6 by activating AKT-ERK-MYC signaling to promote cell dedifferentiation.^300^ Obesity is another important PDAC risk factor.^301–304^ Obesity increases fatty pancreatic infiltration which is correlated with development of pancreatic intraepithelial neoplasia (precursor of PDAC).^305^ While the consumption of alcohol and nicotine has changed only slightly in most developed countries, obesity is increasing rapidly in recent decades and has become an epidemic in many countries.^306^ This explains, at least partially, the rapid increase in PDAC incidence. Germline mutations are found in approximately 10% of PDAC patients.^307^ These patients have a positive family history of PDAC and are usually diagnosed at a relatively young age.^308^ Mutations are commonly found in the genes *BRCA 1/2, PALB2, ATM, CDKN2A, STK11* and the DNA mismatch repair genes *MLH1, MSH2*, and *MSH6*.^307,309,310^ Patients with a family history of PDAC (defined as two or more first-degree relatives diagnosed with PDAC) or who carry one of these germline genetic variants have a significantly increased risk of developing PDAC.^308^ Therefore, PDAC screening is recommended for this group on the basis of their individual risk.^309,310^ In contrast, PDAC screening is not recommended for the general population.^311^

### Molecular pathology and tumor biology

#### Genomic aberrations

The conversion of precancerous lesions to PDAC is the hallmark of carcinogenesis in the pancreas. This transformation is driven by a stepwise accumulation of mutations in key oncogenes and tumor suppressor genes.^312–314^ Four main driver genes have been identified: The oncogene Kirsten rat sarcoma virus (*KRAS*) and the three tumor suppressor genes tumor protein 53 (*TP53*), cyclin-dependent kinase inhibitor 2A (*CDKN2A*), and SMAD family member 4 (*SMAD4*). The presence of a mutation in *KRAS* in particular, but also in *CDKN2A*, is associated with a significantly worse prognosis.^315^ Mutations in *KRAS* are common (>90%) and are usually found early in the process of malignant transformation.^296,314^ KRAS is activated by the binding of GTP and is inactivated by GTP hydrolysis.^313,316^ *KRAS* mutations usually occur as point mutations in codon 12, resulting in KRAS being unable to hydrolyze GTP and therefore remaining activated.^313,316^ KRAS activates the RAF/MEK/ERK and phosphoinositide3-kinase/protein kinase B (PI3K/AKT) signaling pathways with subsequent cell cycle progression and increased cell survival.^313^ Mutations in the critical tumor suppressor gene *TP53* occur in 50-70% of patients and are characteristically found at advanced stages of malignant transformation.^313^ TP53 is activated in response to a variety of cellular stressors, such as oncogenic activation or DNA damage, and triggers multiple cascades such as cell cycle arrest, DNA repair and apoptosis, which is not possible in the case of *TP53* mutation.^299,313^ In addition, multiple rare mutations (usually <10%) are found in the *KDM6A*, *MLL3*, *ARID1A*, *TGFBR2*, and *BRCA1/2* genes, among others.^312–314^

#### Molecular subtypes

Currently, treatment decisions in PDAC are made mainly irrespective of the molecular characteristics of the individual cancer. However, on a molecular level, PDAC is highly variable from patient to patient. In particular, transcriptome profiling studies have shown that PDAC can be divided into several subgroups.^314,315,317–321^

Studies have introduced different nomenclatures, which a recently published phylotranscriptomic tree has attempted to standardize.^315^ The classical and basal (or squamous) molecular subtypes are clearly distinguishable. The classic subtype may be further subdivided into immune classic, pure classic precursor, and ADEX. The COMPASS study was a prospective study designed to investigate how the molecular subtype affects survival and response to chemotherapy.^322,323^ The study showed that survival is significantly worse in the basal (or squamous) subtype (median overall survival 6.3 versus 10.4 months in comparison to the classic subtype). The response to mFOLFIRINOX chemotherapy is also significantly worse in the basal subtype (progression-free survival 2.7 versus 8.5 months in comparison to the classic subtype). Interestingly, the basal subtype was only found in metastatic PDAC, suggesting more aggressive cancer biology in this subtype. GATA6 is a prognostic marker and is highly unregulated in the classical subtype. The usefulness of these molecular subtypes and GATA6 for individualized therapy is currently being investigated in prospective studies (e.g., NCT04469556).

#### Tumor microenvironment

The tumor microenvironment comprises multiple cellular and non-cellular components and is a hallmark of PDAC, influencing malignant transformation, tumor growth, metastasis, and response to therapy.^324,325^ However, the effects of these interactions are often subject to dynamic changes during tumor development and often include tumor-promoting and tumor-suppressing aspects.^326,327^ The tumor microenvironment comprises different cell types, with the most interesting data available for interactions with fibroblasts, peripheral neurons and immune cells. Comprehensive reviews on all aspects of the tumor microenvironment have been published.^328,329^ Histological examination of PDAC shows desmoplasia, which represents extracellular matrix deposition. This dense extracellular matrix is mainly produced by cancer-associated fibroblasts (CAFs) and contains collagen, fibronectin, laminin, and hyaluronic acid. On the one hand, the deposition of extracellular matrix by CAFs leads to an increase in interstitial pressure, which explains the low vascularization and consequent nutrient deprivation.^330^ This results in a nutrient-poor environment that inhibits tumor growth.^331–333^ On the other hand, CAFs secrete nutrients, mediators, and growth factors that promote tumor growth.^334–336^ In addition, the dense extracellular matrix creates a physical barrier that hinders the penetration of chemotherapeutic agents and is probably an important reason for the poor response to different types of chemotherapy.^324,333,337^ The timing of CAF inhibition and which of the different subgroups are inhibited may determine whether the overall outcome is tumor-suppressive or promoting. However, global inhibition of CAFs has not been shown to improve survival in PDAC.^338,339^ In addition, there is a very close interaction between PDAC cells and peripheral nerves.^340^ PDAC cells attract nerves by secreting neurotrophins, particularly nerve growth factors. Through innervation, tumors acquire various growth factors and nutrients.^327^ In a landmark study, PDAC-attracted nerves were shown to provide the cancer with the amino acid serine, which is essential for growth but in short supply due to the nutrient-poor environment.^341^ However, tumor-nerve interactions also have heterogeneous effects on tumor growth. Increased input from sensory and sympathetic nerve fibers promotes malignant transformation and tumor growth, whereas parasympathetic input has the opposite effect.^342–344^ Several studies are currently investigating the benefits of a beta-blocker (inhibition of sympathetic input) or bethanechol, a muscarinic receptor agonist (increasing the parasympathetic input) in PDAC.^327^ Another promising strategy is the inhibition of PDAC-mediated NGF production, which significantly attenuated tumor-nerve interaction in animal models and thus had a significant tumor-suppressing effect.^345^

In addition, alteration of the local immune cells leads to an immunosuppressive tumor environment. This is mainly characterized by an abundance of tumor-associated macrophages (TAMs), myeloid-derived suppressor cells (MDSCs), and regulatory T cells, while T effector cells are rare.^328,329^ Infiltration of TAMs and MDSCs into tumor tissue is associated with reduced survival.^346^ This immunosuppressive tumor microenvironment is thought to be an important reason for the poor response to immunotherapy (see below).

### Current management

Several therapeutic strategies are available for PDAC treatment. Importantly, two parameters are particularly important in determining treatment strategy in clinical practice: tumor localization and fitness of the patient. Firstly, the relationship between PDAC and the surrounding vascular structures and the presence of distant metastases needs to be clarified. This is usually done using a CT scan, which has good diagnostic accuracy and is superior to MRI in showing the spatial relationship of the tumor to the surrounding vascular structures.^347^ Based on this staging, patients can be divided into four groups: Resectable, borderline resectable, locally advanced, and metastatic disease.^348^ Resectable PDAC is defined as having no arterial or venous involvement and no distant spread. Contact with the superior mesenteric artery <180 degrees is considered borderline resectable, while contact >180 degrees is considered locally advanced.^349,350^ Furthermore, contact with the portomesenteric venous axis, celiac axis, hepatic artery, as well as biological and conditional factors must be considered.^351–353^ Secondly, as aggressive chemotherapeutic drugs may be used as antineoplastic therapy (see below), the general condition of patients is a major factor in therapeutic decision-making. This is assessed using the ECOG performance status. In particular, a distinction is made between fit patients (ECOG 0-1) and patients who need help with activities of daily living (ECOG ≥2). Furthermore, with the introduction of targeted therapy that rely on the presence of actionable genetic alterations—as outlined below—genetic testing is recommended by the NCCN guidelines for any patient with confirmed PDAC.^354^ Currently, testing for mutations in the following genes is recommended: *BRCA1/2*, *KRAS*, and *RET/NTRK*. Furthermore, genetic counseling is advised for patients who test positive for a pathogenic mutation in *ATM, BRCA1, BRCA2, CDKN2A, MLH1, MSH2, MSH6, PALB2, PMS2, STK11*, or *TP53*, and for patients with a positive family history of PDAC, regardless of mutation status.

### Management of resectable PDAC

Most patients with PDAC remain asymptomatic for a long time and only develop symptoms in advanced stages. Thus, only about 15% of patients are diagnosed with resectable PDAC.^299^ These patients undergo upfront surgery with adjuvant chemotherapy. Complete resection of the tumor and regional lymph nodes is the goal of surgery, as resection (with adjuvant chemotherapy) is the only curative treatment approach. R0 resection, small tumor size, and tumor-free lymph nodes are the most important predictors of long-term survival.^355^ Adjuvant chemotherapy significantly prolongs overall survival and reduces cancer recurrence.^356,357^ Adjuvant chemotherapy begins 4–8 (maximum 12) weeks after resection, once the patient has recovered from surgery.^358^ The standard of care is a modified FOLFIRINOX (mFOLFIRINOX; 5-fluorouracil, leucovorin, oxaliplatin with reduced irinotecan and without 5-fluorouracil bolus) chemotherapy regimen. In a prospective study, mFOLFIRINOX prolonged disease-free survival from 12.8 months to 21.6 months compared to gemcitabine.^359^ Patients with contraindications to mFOLFIRINOX or those with reduced performance status may receive gemcitabine with or without capecitabine as an alternative treatment of choice.^299,357,360^ The question, if neoadjuvant or perioperative chemo(radio)therapy is beneficial in resectable disease, is an area of active research. Results from ongoing trials are awaited e.g. PREOPANC-3^361^ or Alliance A021806.^349,362^ However, the PANACHE01-PRODIGE48 trial showed no survival benefit of neoadjuvant mFOLFIRINOX or FOLFOX compared with upfront surgery.^363^ Similarly, NORPACT-1 evaluated the benefit of neoadjuvant mFOLFIRINOX in resectable PDAC.^364^ It showed a nonsignificant reduction in median survival with neoadjuvant mFOLFIRINOX versus upfront surgery (25.1 versus 38.5 months).

### Management of borderline resectable and locally advanced PDAC

The management of patients with borderline and locally advanced PDAC can be challenging and usually requires individualized and multidisciplinary decision-making in specialized centers. A major problem is the lack of randomized controlled trials answering important questions in these subgroups. Accordingly, clinical practice guidelines vary.^365–368^ Upfront surgery is considered suboptimal and associated with poorer survival in patients with borderline respectable or locally advanced PDAC, though.^351,369^ Thus, most guidelines favor neoadjuvant treatment with secondary resection. This is mainly supported by three randomized controlled trials of neoadjuvant therapy for borderline resectable PDAC. Neoadjuvant therapy consisted of different radiochemotherapy and chemotherapy regimens, followed by resection and adjuvant chemotherapy. Overall, neoadjuvant therapy significantly improved survival and achieved a higher rate of R0 resection.^370–372^ For example, the ESPAC5 trial showed an improvement in 1-year survival rate of 39% versus 84% comparing upfront surgery with four cycles of neoadjuvant FOLFIRINOX and subsequent resection.^370^ However, due to the small number of cases, the effect was not statistically significant. Randomized controlled trials are underway to determine which neoadjuvant therapy is most effective. Currently, the NCCN guidelines recommend chemotherapy (FOLFIRINOX or gemcitabine with nanoparticle albumin-bound paclitaxel) with or without radiation.^373^ In locally advanced PDAC, primary resection is usually not possible. Treatment decision is, therefore, based primarily on the performance status of the patient (similar to the metastatic situation). Patients with a good performance status (ECOG 0-1) should receive aggressive chemotherapy with mFOLFIRINOX.^299^ There is hope that secondary resectability can be achieved after a good response to treatment.^374^ It is important to remember that locally advanced PDAC is a very heterogeneous group from a surgical perspective. For example, anatomical criteria (localization in head versus tail, venous versus arterial vascular infiltration), response to previous chemotherapy and performance status have a significant impact on whether secondary surgical therapy is possible. There appears to be a wide variation in the literature as to how many patients with locally advanced PDAC receive secondary resection, ranging from 13 to 61%.^375,376^ This must be seen in the light of the continuous improvement in surgical techniques and intensive care in general. For example, some tumors that were previously considered inoperable are now operable due to improvements in venous and arterial reconstruction. However, these data once again underline the importance of ensuring that patients with locally advanced PDAC receive multidisciplinary care in a specialized center. Patients with a moderate performance status (ECOG 2) may benefit from less aggressive chemotherapy in locally advanced PDAC and can receive gemcitabine with or without nanoparticle albumin-bound paclitaxel.^299^ Patients with poor performance status (ECOG ≥3) should receive palliative chemotherapy (e.g., gemcitabine) or best supportive care.^299^

### Management of metastatic PDAC

**Fig. 5:**
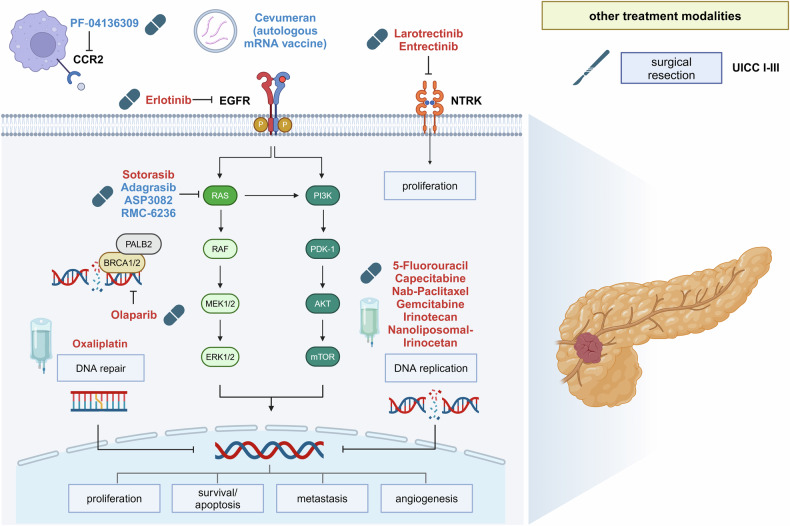
Current therapeutic targets in pancreatic cancer. 5-FU 5-fluorouracil, EGFR epidermal growth factor receptor, NTRK neurotrophic tyrosine receptor kinase. Therapies that are recommended by international guidelines are marked in red. Therapies with preclinical or only low levels of clinical evidence are highlighted in blue

Approximately one in two patients has metastatic disease at the time of diagnosis. At this stage, systemic therapy is indicated to relieve tumor-related symptoms and prolong survival (see Fig. 5). Gemcitabine was the first chemotherapy for which a survival benefit could be shown in metastatic PDAC. Compared with 5-fluorouracil, median survival was increased from 4.4 to 5.6 months.^377^ Although the survival benefit is modest, gemcitabine monotherapy has a very favorable side-effect profile and is, therefore, still the treatment of choice for patients with poor performance status (ECOG ≥2). However, new poly-chemotherapy regimens have been developed that have further improved patient survival. The chemotherapy regimen FOLFIRINOX is a potent chemotherapy regimen that has been shown to improve overall survival from 6.8 months to 11.1 months compared with gemcitabine monotherapy.^359^ The adapted mFOLFIRINOX regimen appears to be equally effective and is frequently used in routine treatment.^378,379^ However, these chemotherapies are associated with a high amount of toxicity and should only be used if the patient is in good general health (ECOG 0-1).^373^ In another phase III trial, the combination of gemcitabine with nanoparticle albumin-bound paclitaxel proved superior to gemcitabine monotherapy. Median overall survival was 8.5 months in the nab-paclitaxel-gemcitabine group as compared with 6.7 months in the gemcitabine group.^380^ Toxicities were higher than with gemcitabine monotherapy, but (in comparison of the arms in two trials) slightly lower than with the FOLFIRINOX regimen.^373,380^ More recently, the NAPOLI3 trial showed that treatment with the NALIRIFOX regimen (liposomal irinotecan, oxaliplatin, leucovorin and fluorouracil) had a significant survival benefit compared with nab-paclitaxel-gemcitabine (overall survival 11.1 versus 9.2 months) and could therefore be considered as an alternative to FOLFIRINOX in the first-line treatment.^381^ A head-to-head comparison between FOLFIRINOX and NALIRIFOX is not yet available. EGFR inhibitor erlotinib in combination with gemcitabine is also approved for first-line treatment of PDAC after showing survival benefit against gemcitabine monotherapy.^382^ However, as more potent combination therapies have been approved since then, this regimen only plays a minor role in clinical practice today, while FOLFIRINOX will probably continue to be most frequently used as first-line treatment in patients with good performance status. Interestingly, meta-analyses have shown that patients with a good performance status benefit greatly from these aggressive poly-chemotherapy regimens, but not those with a poor performance status (ECOG ≥2).^383^ These patients should be treated with gemcitabine monotherapy.^373^ For patients who progress on gemcitabine-based therapies, nanoliposomal irinotecan plus 5-fluorouracil is a second-line treatment option. In the phase III NAPOLI-1 study, this combination showed a survival benefit compared with 5-FU (overall survival 6.1 versus 4.2 months).^384^ For patients who progress on FOLFIRINOX, gemcitabine with or without nanoparticle albumin-bound paclitaxel represents the second-line treatment of choice.^299,373^

#### Advanced or metastatic BRCA1/2 mutated PDAC

Mutations in homologous recombination repair genes, including *BRCA1*, *BRCA2*, and *PALB2*, lead to compromised DNA repair capacity and genomic instability. Poly(ADP-ribose) polymerase (PARP) is required to repair DNA single- and double-strand breaks.^385^ Interestingly, neither BRCA mutation alone nor PARP inhibitors alone are lethal, but the combination is.^385^ Furthermore, platinum-based chemotherapies lead to DNA cross-linking and thus inhibit DNA synthesis. The use of PARP inhibitors or platinum-based chemotherapy in homologous recombination repair-deficient PDAC appears to be promising due to an accumulation of DNA damage ultimately leading to tumor cell apoptosis.^386–388^ In addition, response to platinum-based therapy (defined as a response or stable disease) is a predictive parameter for response to PARP inhibition. The PARP inhibitor olaparib is the first biomarker-based targeted therapy approved for metastatic PDAC. Patients with mutated *BRCA1* or *BRCA2* who have not progressed after at least 16 weeks of first-line platinum-based chemotherapy are eligible for olaparib maintenance therapy.^388^ Compared to placebo, olaparib improved median progression-free survival from 3.8 to 7.4 months, although no difference in overall survival was observed.^388^ Based on these promising data, further trials are underway, such as the APOLLO trial^389^, which is investigating the use of PARP inhibitors in the adjuvant setting for resectable PDAC with *BRCA1*, *BRCA2*, or *PALB2* mutations. Preclinical models and studies of ovarian cancer have shown that homologous recombination repair-deficient tumors may respond well to a combination of PARP inhibitors and an immune checkpoint inhibitor.^390,391^ Interestingly, immune checkpoint inhibition with anti-CTLA-4 has been shown to be superior to PD1/PDL-1 inhibitors in this setting.^390,392^ In patients with locally advanced or metastatic PDAC, the combination of PARP inhibitor with anti-CTLA-4 antibody was shown to have a significantly higher six-month progression-free survival (59.6%) than PARP inhibitor with anti-PD1 antibody (20.6%).^392^ Further studies are ongoing to investigate the benefit of combining PARP inhibitors with checkpoint inhibitors as maintenance therapy in homologous recombination repair-deficient PDAC (NCT04666740).^393^

### Clinical research progress

#### Early detection

One of the main reasons for the poor survival of PDAC is the fact that over 50% of patients are first diagnosed at a metastatic stage, which is associated with a poor prognosis. To this end, the question arises how earlier detection can be achieved. The most obvious answer is a screening program, but these are considered inappropriate for the general population due to a poor risk-benefit ratio. Currently, screening is only recommended in high-risk populations, defined as those with a familial PDAC or carrying known risk genes.^309,310^ However, only about 10% of PDAC mutations are germline mutations. This means that the majority of PDAC cases are not eligible for screening programs. To identify groups of people at increased risk of developing PDAC due to somatic mutations, various strategies have been discussed. These strategies range from simple scores to recently presented AI algorithms that can identify a risk group based on the sequence of diagnostic codes. This AI approach is able to detect people with a >100-fold increased risk of developing PDAC within 3 years compared to the general population by analyzing their disease trajectories.^394,395^ If such approaches can be validated, it would certainly be possible to conduct targeted screening in these identified risk groups. In line with this, a deep learning approach (PANDA) has recently been used to show how pancreatic lesions can be detected and classified with high accuracy using non-contrast-enhanced CT.^396^ Furthermore, there are many other approaches, essentially based on blood tests. Several blood/serum components (classical tumor markers such as CA19-9, circulating proteins, circulating tumor cells, circulating cell-free nucleic acids, and circulating exosomes) were analyzed, but largely failed to show promising results as a screening tool.^395^ Currently, the most promising approach is probably the study of circulating exosomes. Exosomes are secreted membrane-enclosed vesicles that carry information about intercellular communication. Melo et al. found that cancer cell-derived exosomes express a specific cell surface proteoglycan, glypican-1.^397^ In their validation cohort, the authors reported 100% specificity and 100% sensitivity in distinguishing PDAC from healthy controls and benign pancreatic lesions. This method is likely to be the best technique currently available for the early diagnosis of PDAC. A problem for implementation in a large population screening is the high technical requirements to isolate glypican-positive exosomes and the fact that glypican-1-positive exosomes are also found in other cancer entities.^395,398^

#### Precision medicine approaches

Molecular differentiation of PDAC has played a subordinate role in the treatment of PDAC, largely because it has had little therapeutic relevance until recently. Even when mutational analysis was performed, it was rarely used in practice to guide treatment decisions (partly due to a lack of clinical trials). In the Know Your Tumor registry, for example, 26% of the 1082 samples analyzed were found to have a targetable mutation.^399,400^ In practice, however, only 2% received a tailored therapy.^400^ Nevertheless, personalized therapy is a great hope for future treatment strategies.

*KRAS* is mutated in most patients and plays a key role in driving PDAC. Historically, pan-KRAS inhibitors were developed, but these failed to demonstrate clinical benefit and led to KRAS being considered ‘untargetable’ for decades.^401^ In most cases, the *KRAS* mutation is a point mutation in codon 12, resulting in G12D, G12V or G12C mutations. As a result, mutation-specific therapeutic strategies have been developed. The lack of classical KRAS binding sites has been one of the main problems, which was at least partially alleviated by the discovery of a drug pocket in KRAS G12C.^402^ Although KRAS G12C affects only 1–3% of patients with PDAC, several small molecule inhibitors targeting KRAS G12C (sotorasib, adagrasib, D-1553, JAB-21882) have been developed (primarily for lung cancer, where KRAS G12C is more commonly found).^403^ In a phase I/II trial, sotorasib demonstrated a therapeutic response in the presence of a KRAS G12C mutation and thus represents a new form of therapy for this subgroup.^404^ This success has also raised hopes of a clinical benefit of other KRAS mutation-specific inhibitors, targeting mutations such as the much more common KRAS G12D and KRAS G12V genotypes (prevalence of 30-40%, each).^403^ A phase I trial of a small molecule specific for KRAS G12D has recently started, following promising in vivo results (NCT04111458).^405^ Finally, the first pan-RAS inhibitor (RMC-6236) was recently developed and showed promising results in a preliminary analysis, leading to the initiation of a phase I trial (NCT05382559). In addition, inhibition upstream of KRAS has also shown promising initial results with the targets SHP2 (Src homology-2 domain-containing protein tyrosine phosphatase-2) and SOS1 (Son of Sevenless 1), which are now investigated in clinical trials (NCT05288205, NCT03634982, NCT05379985).^406^ In addition, there are rare mutations (usually <1–5% of all PDAC cases) that influence the oncogenesis of PDAC. Drugs targeting these rare alterations are usually investigated in basket studies, which have also included a small number of PDAC patients. Based on these studies, some inhibitors targeting rare mutations have already received FDA approval for solid tumors. *RET* (rearranged during transfection) fusions are most commonly found in thyroid cancer, whereas in PDAC they occur in <1% of all cases.^236^ *RET* fusion leads to permanent, ligand-independent activation of the RET pathway.^236^ Selpercatinib is a selective RET kinase inhibitor and has also been used in a basket study in 12 patients with PDAC.^236^ PDAC patients showed a response rate of 55% representing a higher response rate compared to other tumor entities. Tumor responses were assessable in eleven patients, with six patients achieving partial response, making selpercatinib a promising option for patients with RET fusion.^373^ In addition, in solid tumors with neurotrophin receptor kinase (*NTRK*) gene fusions, larotrectinib, and entrectinib are inhibiting NTRK and both showed benefits in basket trials, leading to their FDA approval.^407–409^ For example, entrectinib showed a partial response in two out of three patients with PDAC.^407^

#### Targeting tumor microenvironment and immunotherapy

The introduction of checkpoint inhibitors has been a milestone in the treatment of many tumor types. However, this is not the case for PDAC. Randomized clinical trials have shown no significant benefit from checkpoint blockade in an unselected cohort.^410^ KEYNOTE-028 was a basket trial in which PD-L1 positive tumors were treated with the anti-PD1 antibody pembrolizumab.^411^ Unfortunately, PDAC showed the lowest response rate of all tumor types studied. However, at least some subgroups of patients with DNA mismatch repair deficiency seem to have a benefit from checkpoint inhibition.^412^ For example, in a basket trial, six patients with DNA mismatch repair deficiency were treated with pembrolizumab and two patients experienced a complete response.^413^ Based on these data, the NCCN guidelines recommend the use of pembrolizumab in metastatic PDAC in the setting of mismatch repair deficiency, microsatellite instability-high or high mutation burden >10 mut/Mb after FDA approval in 2017.^373^. The tumor microenvironment is thought to be a major contributor to poor response to checkpoint inhibitors. Various parts of the tumor microenvironment have therefore been inhibited with modest success in several trials.^339,414^ However, there are promising data for inhibition of the chemokine receptor CCR2 (C-C chemokine receptor type 2), which prevents PDAC-induced recruitment of MDSCs. In a phase I study, the CCR2 antagonist PF-04136309 was evaluated in combination with FOLFIRINOX versus FOLFIRINOX. The combination showed an improved objective response rate of 49 versus 0%.^346^ However, the administration of PF-04136309 together with gemcitabine/nab-paclitaxel did not result in a better response rate than the administration of gemcitabine/nab-paclitaxel alone, but increased the rate of pulmonary toxicities.^415^ There are other promising approaches in this area. Autologous cevumeran (an individualized mRNA vaccine) was tested in 16 patients with resectable PDAC.^416^ This treatment showed successful vaccine-induced expansion of T cells (vaccine responders) in 50% of patients. At a median follow-up of 18 months, 100% of vaccine responders were relapse-free, while the median relapse-free survival in the non-responder group was 13.1 months. A phase II trial in resectable PDAC has recently started (NCT05968326). The benefits of autologous cevumeran are also being investigated in clinical trials for locally advanced and metastatic PDAC (NCT03289962). Recently, the potential of mRNA vaccines for the reduction of tumor recurrence was shown. Three-year follow-up data from a phase I trial of an individualized mRNA vaccine in patients with surgically removed pancreatic ductal adenocarcinoma continue to show polyspecific T-cell responses up to 3 years after vaccination and a strong reduction of risk for relapse.^416^ Based on these results, a phase II study is ongoing (NCT05968326).

Apart from the PDAC—immune cell interaction, preclinical data highlighted the importance of PDAC – nerve interaction in particular. Thus, neurons provide PDAC with nutrition and growth factors and seem to play an important role in the development of PDAC-induced abdominal pain.^341,345,417^ However, our understanding of it is not yet advanced enough to translate this into clinical trials.

Another important strategy is the combination of different therapeutic approaches. A good example is the combination of the CCR2 antagonist PF-04136309 with FOLFIRINOX, which is very effective, whereas the combination with gemcitabine/nab-paclitaxel (see above) did not add any benefit.^346,415^ Hence, rational design of effective combination therapies remains challenging and will require extensive clinical testing.

## Colorectal cancer

### Epidemiology

Colorectal cancer (CRC) is the third most common cancer worldwide and the second leading cause for cancer-related death.^2^ Around 1.9 million new cases were diagnosed with the disease in 2022, and more than 903,000 patients died from it in that year.^2^ The burden of disease is highest in developed countries, particularly in Australia and Eastern Europe and lowest in Africa and Southern Asia.^418^ While incidence rates are increasing in developing regions, Western countries have seen a decrease in the incidence and mortality rates during the last 20 years, mainly due to better prevention, early detection, and improved treatment strategies. However, this decline slowed down recently because of a marked increase of cases observed in younger adults.^419^ This trend seems to be associated with changing lifestyle and environmental factors, including known risk factors such as obesity, lack of physical activity, a diet rich in processed food and red meat, as well as increased alcohol intake. Further risk factors for CRC include having inflammatory bowel disease, a family history of CRC and hereditary conditions, such as familial adenomatous polyposis syndrome (FAP) or hereditary non-polyposis CRC (HNPCC).

### Molecular pathology and tumor biology

Colorectal cancer develops gradually, forming benign precursor lesions (adenoma-carcinoma sequence).^420^ At the molecular level, mutations in tumor suppressor genes such as *APC* (adenomatosis polyposis coli) and *TP53*, as well as chromosomal instability with changes in gene copy numbers and translocations, are often found.^421^ Of particular relevance for therapy is the less common subtype with defective mismatch repair system (dMMR), characterized by the presence of numerous point mutations (hypermutated tumors) and microsatellite instability (MSI). This occurs in approximately 5–10% of cases and is associated with a response to immune checkpoint inhibitors (see below).^422^ In addition, frequent RAS mutations (approximately 50%) and *BRAF* mutations (~5–10%) are therapeutically relevant in the metastatic stage. Based on RNA expression data, CRC can also be classified into different molecular subtypes and out of these classification systems, four consensus subtypes have been established: In addition to the abovementioned “MSI/immune” subtype, there is a “canonical” subtype related to high WNT signaling pathway levels, a “metabolic” subtype with dysregulated metabolic pathways and a “mesenchymal” subtype characterized by epithelial-mesenchymal-transition and a high amount of stromal fibroblasts.^423^ The clinical utility of this molecular classification remains controversial, though. Thus, with respect to molecular diagnostics, analysis of MMR / MSI status is mandatory in all cases that might require medical therapy (≥ stage II). In patients with metastatic or unresectable disease, additionally, the analysis of RAS and *BRAF* mutation status is required. Further molecular diagnostics (HER2 overexpression, panel sequencing) is done in therapy refractory cases.

### Current management

The therapy for CRC continues to be primarily based on the UICC/TNM stage and the tumor location, i.e. anatomical and microscopic extent of the disease. In addition to further predictive and prognostic factors, the patient’s general condition and age play important roles in treatment decisions.^424^

### Management of early-stage CRC

Tumors in stage I (T1/2 N0 M0) are confined to the submucosa or muscularis propria and do not have lymph nodes or distant metastases. These tumors are usually treated surgically or by endoscopic removal and have an excellent prognosis with 5-year survival rates of over 90%. Adjuvant therapy is not performed. After endoscopic removal of a presumed adenoma^425^, histological examination sometimes reveals an invasive carcinoma. In T1 tumors histopathologic features predict low risk of lymph node metastasis (R0, G1-2, L0, no tumor budding) so that surgical resection can be avoided in these cases.^426^

In stage II (T3-4 N0 M0), characterized by tumor invasion beyond the muscularis propria, and in lymph node-positive stage III (T1-4 N1-2 M0), patients with colon carcinoma are primarily treated surgically. Adjuvant chemotherapy is not necessary for most patients in stage II: The largest published study on adjuvant therapy in stage II (QUASAR) showed an improvement with chemotherapy compared to surgery alone, but the benefit was only 3.6% in 5-year survival rates.^427^ Additionally, this study had methodological weaknesses, and further (smaller) studies could not show a significant survival advantage with chemotherapy in stage II. Since certain risk factors markedly worsen the prognosis of stage II colon cancer, it is assumed that the presence of these factors indicates a higher survival benefit from adjuvant therapy compared to low-risk situations. For example, the 5-year survival rate for some patients in stage II (e.g., stage IIb/c with T4 depth of invasion) is worse than in stage IIIA (T1 N2a or T1/2 N1), but a benefit of adjuvant therapy is established in stage III (see below). Therefore, adjuvant chemotherapy may be administered in stage II disease with prognostically unfavorable factors. Established risk factors include T4 classification (especially with perforation) and <12 examined lymph nodes. Other risk factors to consider are emergency surgery, invasion of blood or lymph vessels, perineural invasion, low tumor differentiation, luminal obstruction, or high preoperative CEA levels.^426^ Recent studies also suggest that the detection of circulating tumor DNA may be useful for determining the indication for adjuvant therapy in stage II CRC: Tie et al. for instance showed that a measurement of postoperative ctDNA to guide adjuvant treatment could reduce the application of adjuvant chemotherapy without compromising recurrence-free survival in the first randomized phase II study (DYNAMIC) on this topic.^428^ Results of further (randomized) trials are pending - these may determine if standard-of-care use of a liquid biopsy test in this setting should be implemented.^429^ The presence of microsatellite instability is associated with better survival in stage II, while adjuvant chemotherapy in these patients has been shown to worsen survival in retrospective analyses, thus, no adjuvant therapy is performed in these cases.^430,431^

### Management of lymph node-positive colon cancer

For lymph node-positive colon carcinomas (UICC stage III, T1-4, N1-2, M0), surgical treatment followed by adjuvant chemotherapy is the current standard therapy. Unlike in stage II, positive clinical trials for adjuvant therapy exist, with an expected benefit of 15–20% in 5-year survival rates. A combination therapy of 5-FU and oxaliplatin is used as a regimen. While a treatment duration of 6 months has been the standard-of-care for a long time, analysis of over 12,000 patients as part of the international IDEA initiative has shown that a 3-month therapy with CAPOX is preferable for most patients.^432^ Although non-inferiority of 3 versus 6 months could statistically not be demonstrated, the difference was only 0.4% in disease-free survival. Given the often irreversible adverse effects of longer oxaliplatin therapy (neuropathy), 3 months is advantageous for most patients, with a stratification based on risk factors: patients at higher risk for recurrence and cancer-associated mortality, defined by the presence of T4 depth of invasion or extensive lymph node metastasis (N2), may still receive therapy for 6 months, while patients at low risk (T1-3, N1) are treated for only 3 months, preferably with CAPOX. Alternatively, neoadjuvant concepts could be increasingly used in colon cancer in the future. For instance, data from the FOxTROT study showed a benefit in 2-year recurrence-free survival with a 6-week neoadjuvant therapy with FOLFOX compared to direct surgery.^433^ These options may be further explored, however, clear detection of lymph node-positive colon cancer by radiological imaging is challenging.

### Neoadjuvant therapy of rectal cancer (stage II-III)

For rectal cancer, precise local staging through endoscopy and quality-assured MRI are necessary for treatment planning. The MRI assesses the distance of the tumor to the mesorectal fascia (MRF), or the future mesorectal resection margin, as well as potentially involved lymph nodes and extramural venous invasion (EMVI). Rectal cancer in stage II/III in the upper third (10–16 m from the anal verge) is treated similarly to colon cancer with surgery and possibly adjuvant chemotherapy. For T3-4 or N+ tumors and tumors located in the lower two-thirds (0–12 cm) in MRI/endosonography, preoperative therapy is indicated. Traditionally, this is done by radiochemotherapy with a total dose of 50.4 Gy combined with 5-FU or capecitabine, or by short-course radiotherapy with 5 × 5 Gy, especially if tumor downsizing before surgery is not necessary. After chemoradiation or radiotherapy, surgery is performed with total mesorectal excision, followed by adjuvant chemotherapy. A new treatment concept is total neoadjuvant therapy (TNT), especially for advanced tumors (T4, N2, MRF+, EMVI). To this end, the RAPIDO trial tested the addition of consolidation chemotherapy with FOLFOX (nine cycles) or CAPOX (6 cycles) to preoperative radiotherapy (5 × 5) and surgical resection against the standard chemoradiation with 50.4 Gy and capecitabine, followed by surgical resection and adjuvant chemotherapy in this setting. Disease-related treatment failure rate was significantly reduced, and the rate of complete responders was increased (14% versus 28%).^434^ The PRODIGE23 study also found significantly improved disease-free survival and response rates with a TNT concept using slightly different protocols. Thus, for patients with high-risk tumors in good general condition, TNT is already the standard, even allowing a close watch-and-wait approach with organ preservation and avoidance of surgery.

### Management of metastatic CRC (stage IV)

**Fig. 6:**
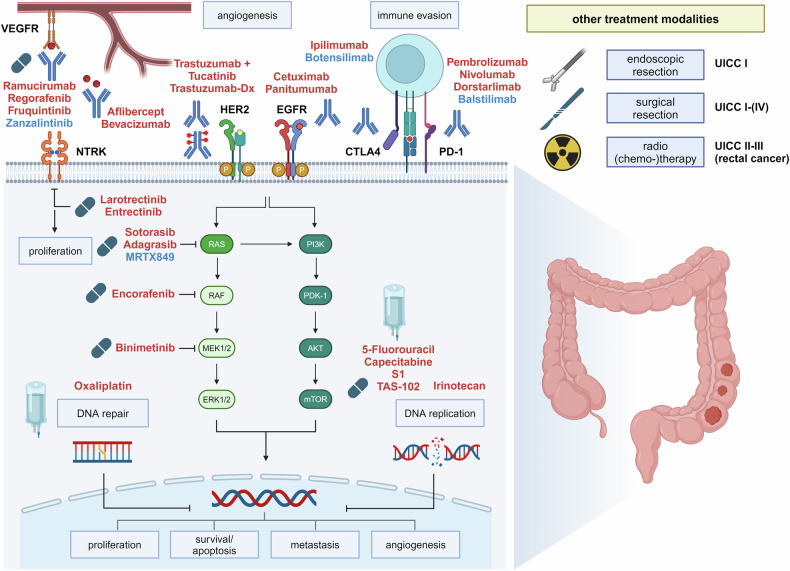
Current therapeutic targets in colorectal cancer. 5-FU 5-fluorouracil, VEGFR vascular endothelial growth factor receptor, HER2 human epidermal growth factor receptor 2, EGFR epidermal growth factor receptor, PD-1 programmed cell death protein 1, PD-L1 programmed cell death 1 ligand 1, CTLA-4 cytotoxic T-lymphocyte-associated protein 4, NTRK neurotrophic tyrosine receptor kinase. Therapies that are recommended by international guidelines are marked in red. Therapies with preclinical or only low levels of clinical evidence are highlighted in blue

Due to increased therapeutic options and improved treatment algorithms, the survival of patients with metastatic CRC has improved in recent years, with studies reporting survival rates of over 30 months (see Fig. 6). The general condition (suitable for intensive therapy?) and the extent of the disease (resectable or potentially resectable metastases?) are crucial for treatment decisions in metastatic CRC. Additionally, tumor biology with predictive factors plays a role in the precise planning of drug therapy.^435^

**Fig. 7:**
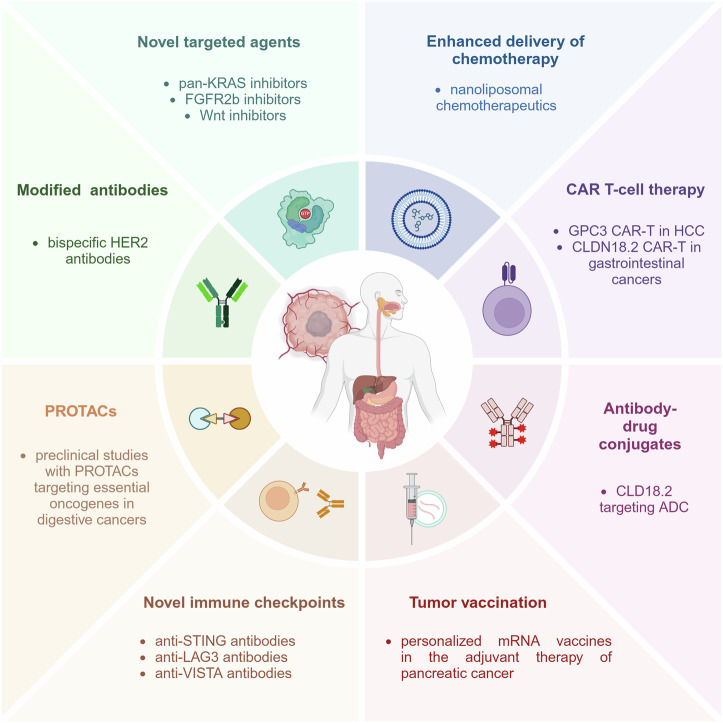
Novel therapeutic developments in digestive cancers. A selection of therapeutic development as well as specific application cases are shown. ADC antibody-drug conjugates, HER2 human epidermal growth factor receptor 2, CLD18.2 claudin 18.2, STING stimulator of interferon genes, LAG3 lymphocyte-activation gene 3, VISTA V-domain Ig suppressor of T cell activation, PROTAC proteolysis-targeting chimera, FGFR2b fibroblast growth factor receptor 2b, GPC3 glypican-3

Patients in good general condition with primarily resectable metastases undergo surgical treatment. After liver resection of CRC metastases, the 5-year survival rate is ~35%. Of these patients, about one-third experience cancer-related death within the next 5 years, while the remaining patients are considered cured.^436^ Patients with non-resectable metastases in sufficiently good general condition initially receive medical therapy Figs. 6, 7.

#### Chemo- and antibody therapy for metastatic colorectal cancer

Fluoropyrimidines have been the cornerstone of drug therapy for CRC for many years, alongside the topoisomerase inhibitor irinotecan^437,438^ and the platinum derivative oxaliplatin^439,440^, which have been used for more than 20 years. These substances are typically used as doublets (FOLFOX/CAPOX or FOLFIRI) in first and second-line treatment.^441^ Both combinations are approximately equally effective in first-line treatment, but differ in their spectrum of side effects.^442^ While irinotecan more commonly causes gastrointestinal side effects (diarrhea), the administration of oxaliplatin is associated with cumulative peripheral neuropathy, which often limits its use. In situations with pressure for remission in patients in good general condition, a triplet (FOLFOXIRI) may also be used).^443,444^ Another survival advantage has been demonstrated through the combination with antibodies against EGFR (cetuximab, panitumumab)^445–447^ or VEGF (bevacizumab, aflibercept, ramucirumab)^448–452^ in first- and second-line therapy. The use of these substances depends on predictive factors. For EGFR antibodies, a negative association of response and survival with the presence of activating mutations in RAS genes has been demonstrated.^453^ The RAS and RAF proteins act downstream of the EGF receptor in the MAPK signaling pathway, so blocking the receptor by therapeutic antibodies is ineffective in the presence of an activating mutation. Another peculiarity of tumor biology is the influence of primary tumor location on prognosis and response to targeted therapies. Patients with metastatic tumors with a right-sided primary site generally have a worse prognosis and respond poorly to therapy with EGFR antibodies.^454^

As a result, first-line therapy for most patients involves treatment with a doublet of fluoropyrimidine and irinotecan or oxaliplatin. In RAS/RAF wild-type tumors and left-sided primary tumor origin, this is combined with an EGFR antibody. For RAS/RAF mutation or right-sided primary tumors, the VEGF antibody bevacizumab is used. There is currently no biomarker for bevacizumab response, although several have been investigated.^455,456^ For older patients and those with reduced general condition, less toxic regimens have been analyzed. Specifically, 5-FU (or capecitabine) in combination with bevacizumab is an effective and well-tolerated regimen for this purpose.^457^ After progression under first-line therapy, second-line treatment follows with the therapy that was not previously administered: FOLFOX is followed by FOLFIRI and vice versa, and EGFR antibodies are followed by VEGF antibodies.^458^ In the case of using a VEGF antibody in the first-line (RAS mutated patients or right-sided primary tumor), the same strategy can be continued beyond progression.^459^

In the third line, TAS-102 (trifluridine/tipiracil) represents an orally available therapeutic option^460^. Data from the SUNLIGHT^460^ study have recently established its combination with bevacizumab in the third line as a new standard. Furthermore, re-exposure to the first-line regimen (especially in cases of initial good response and a longer interval since the last administration) is also common practice.^461^ If EGFR antibody rechallenge is planned, liquid biopsy tests have been proposed to rule out secondary RAS or RAF mutations, as they may be acquired during EGFR antibody therapy and diminish efficacy of EGFR antibody rechallenge.^462^ In refractory tumors, the tyrosine kinase inhibitor fruquintinib was recently approved after positive data from the FRESCO-2 study.^463^ Additionally, regorafenib is also approved for refractory disease.^464^

#### Metastatic MSI/dMMR positive colorectal cancers

A special case are MSI-positive tumors, which represent about 5% of cases in the metastatic stage. The high number of neoepitopes generated in hypermutated tumors and a high degree of lymphocytic immune cell infiltration are assumed to be the reasons for the high effectiveness of checkpoint inhibitor treatment in this subgroup of tumors. These therapies lead to the disinhibition of the immune system through the blockade of the PD1/PD-L1 or CTLA-4 systems, resulting in T-cell-mediated recognition and destruction of tumor cells. The study by Le et al. ^422^ in 2015 first demonstrated proof-of-principle in MSI tumors (40% response rate in pretreated MSI tumors versus 0% in microsatellite-stable (MSS) tumors), leading to the approval of pembrolizumab as the first “morphology-agnostic” drug specifically for MSI. In the phase III KEYNOTE-177 study, pembrolizumab doubled the progression-free survival in first-line treatment to 16.5 months (versus 8.3 months with standard therapy) with significantly lower toxicity.^465^

#### Metastatic BRAF V600E mutant colorectal cancers

A *BRAF V600E* mutation is found in approximately 5% of metastatic CRC. These tumors exhibit a particularly aggressive phenotype, respond poorly to chemotherapy, and have a worse prognosis. However, there is an association between BRAF mutation and MSI, providing a good treatment option for some patients. For other patients, first-line therapy initially involves chemotherapy with the addition of bevacizumab. Contrary to earlier beliefs, intensive triplet therapy has not proven to offer a specific survival advantage for *BRAF*-mutated tumors.^466^ Therefore, FOLFOX with bevacizumab is considered the standard of care for these tumors. In second-line treatment of *BRAF V600E* mutated tumors, a targeted therapy combination with the BRAF V600E inhibitor encorafenib and the EGFR antibody cetuximab has been established by the BEACON study, finding a significantly prolonged progression-free survival, compared to the control group (FOLFIRI or irinotecan with cetuximab).^467^ The additional EGFR antibody targets a resistance mechanism via a feedback loop, which renders monotherapy ineffective in CRC with BRAF inhibition.^468^

### Clinical research progress

The therapy of CRC has continued to improve in recent years. In the adjuvant setting, we have learned that most patients only need 3 months of chemotherapy and thus face a very good prognosis with less toxicity. (Total) neoadjuvant concepts are also on the rise. In the metastatic setting, most patients now have a median survival of >30 months through chemotherapy and antibody therapy. Surgery for metastases even offers a chance of cure for some patients. Patients with microsatellite instability tumors treated with immune checkpoint inhibitors also have a chance of long-term survival. In addition, there are individual therapies, such as those against BRAF mutations, that directly target driver mutations. However, most patients with metastatic disease still cannot be cured. Currently, major research frontiers thus include precision oncology and targeted therapies, as well as advancements in immunotherapy, with MSS/pMMR (immunologically “cold”) tumors as a specific hurdle.

#### Precision medicine approaches in CRC

Precision medicine has the goal to identify treatments for individual patients based on molecular profiling mutations or other molecular characteristics of the disease. To this end, a few recurrent targetable alterations can be detected in CRC, however, at low frequencies.

One possible option for RAS wild-type tumors may be the use of targeted therapies against HER2. HER2 overexpression is rare in CRC (2–3%) but may also occur as a secondary resistance mechanism to anti-EGFR therapy. This was initially analyzed in xenograft mouse models^469^ and later confirmed by the same research group in humans through the HERAKLES study.^470^ More recently, in the multicenter, phase II Mountaineer study, small molecule HER2 inhibitor tucatinib in combination with trastuzumab showed meaningful anti-tumor activity for chemotherapy-refractory, HER2-positive, RAS wild-type unresectable or metastatic CRC and became the first FDA approved HER2 regimen in CRC.^471^ Newer antibody-drug conjugates, such as trastuzumab-deruxtecan, also showed promising activity in early-phase studies.^472^ Interesting early data also exist for the new, mutation-specific KRAS inhibitors such as adagrasib^473^ or sotorasib^474^, which have shown promising disease control rates for G12C mutations (however, only present in 3% of CRC). A combination of cetuximab and adagrasib appears to improve efficacy (median response of more than 6 months in pretreated tumors).^475^ Other inhibitors, also targeting more common variants such as G12D and non-specific (K)RAS inhibitors, are currently in preclinical and early clinical development.^476^ Other potential targets in the RAS/RAF pathway, such as MEK or ERK or in the PI3K/mTOR signaling pathway (including IGF, PIK3CA, mTOR) have so far not been very successfully targeted in clinical studies. Very rarely (<1%), *NTRK* fusions can be detected in colorectal carcinomas. Effective drugs (entrectinib, larotrectinib) are available and approved for these fusions.^407,408^ Other gene fusions are similarly rare or even rarer in CRC. *ALK* fusions, for instance, are known drivers and druggable targets in lung cancer. Case reports of ALK tyrosine kinase inhibitors used in *ALK* fusion positive CRC show promise^477^, so that diagnostic gene sequencing panels used in refractory disease should also include *NTRK*, *ALK* and other known fusion genes (e.g., *ROS*, *RET*). An association of these cancer-driving gene fusions with MSI has been reported. Gene fusions may occur in up to 12% in these cases^478^, particularly in RAS/RAF wild-type disease, so that testing may be particularly worthwhile in these tumors.

#### Immunotherapy advances in CRC

Immune checkpoint inhibitors have vastly improved cancer therapy. In CRC, they are the standard of care in MSI/dMMR metastatic or unresectable disease. In this setting with already good success rates, current studies investigate biomarkers for response/resistance, as well as combination therapies to further enhance efficacy. For instance, PD-1 antibody nivolumab in combination with CTLA-4 antibody ipilimumab showed a 2-year overall survival rate of 78% in a phase II study.^479^ Additional studies, also combining checkpoint inhibitors with chemotherapies and targeted therapies, are underway.

Immune checkpoint inhibition is also investigated in non-metastatic MSI/dMMR tumors. For instance, immune checkpoint inhibition in MSI-positive patients with locally advanced rectal cancer is a very promising emerging option. A seminal study by Cercek et al. ^480^ reported data from 12 evaluable patients within a phase II study, all of whom showed complete remission after treatment with the checkpoint inhibitor dostarlimab. Although follow-up of these patients is still short and the number of cases is limited (MSI-high status is rare in rectal cancer), the data illustrates the potential for surgery-free cure in this subgroup. In colon cancer, investigators of the NICHE study tested a neoadjuvant application of ipilimumab and nivolumab (1 and 2 doses, respectively) before resection of colon cancer in 20 MSI and 21 MSS patients. All the MSI patients had a pathological response, 19 of 20 a major pathological response. Even MSS tumors responded in 27%. Preliminary data from the NICE2 study confirmed this data: All 112 patients in this study responded, 95% had a major pathologic response, 67% a complete pathologic response, which may prompt organ sparing approaches in the future, for instance supported by circulating tumor DNA analyses or novel imaging techniques.^481^

In metastatic disease, immunotherapy with checkpoint inhibitors is not effective in MSS CRC. Making the immunologically “cold” tumors “hot” (and thus treatable) is currently the subject of numerous research projects. Patients with non-liver metastatic CRC might have a better response to immunotherapies, however, further research is needed to define underlying mechanisms and potential therapeutic exploitation. Several trials have been using combination therapies of checkpoint inhibitors with antibodies or targeted therapies, e.g., against VEGF or members of the RAS/MAPK pathway, as well as DNA damage response pathways, to increase immunogenicity. Even though some early-phase studies have been positive, no breakthrough results have been reported so far, though.^424^ Other strategies are based on further developing the immunotherapeutic antibodies. To this end, phase I data on the modified CTLA-4 antibody botensilimab^482^ were recently reported with some long-lasting remissions in combination with a PD-L1 antibody in MSS metastatic colorectal carcinomas.^483^ Larger studies and further developments in this area are expected in the coming years. Additionally, new immune checkpoint targets are being identified, such as VISTA, TIGIT, LAG3, and STING signal pathways that are currently investigated as therapeutic targets.^484^ A further strategy to boost the immune system in immunotherapy approaches is the modulation of the gut microbiome. In general, understanding the complex interplay between the gut microbiome and CRC development, progression, and treatment response has become an important research topic. Investigating the potential of modulating the gut microbiota composition as a therapeutic strategy, such as through probiotics, prebiotics, and fecal microbiota transplantation for different cancers, including CRC, is currently a promising strategy investigated in several studies, especially within immunotherapy concepts.^485^

Thus, the treatment landscape of CRC is under continuous development due to a high number of clinical and translational research efforts, so that many new developments are expected in the coming years, especially in the fields of precision medicine, immunotherapies, and microbiome research.

## Perspectives

In the last decade, in-depth molecular and genetic characterization of many cancers of the digestive systems have provided a deeper understanding of their tumor biology, identifying genetic and non-genetic aberrations that drive carcinogenesis and disease progression. In particular, major progress in the field of immuno-oncology resulted in the wide-spread introduction of immune checkpoint inhibitors in the treatment of nearly all digestive cancers. On the other hand, most tumor-specific molecular alterations that were discovered by multi-omics characterization are not yet actionable or only present at very low frequencies in most cancer entities, e.g., NTRK fusions. Thus, chemotherapy remains the main pillar of systemic therapy for most advanced cancers of the digestive systems and tumor entities that offer a broader spectrum of molecular targets, such as CCA or GC, remain the exception. In the final chapter, we discuss current challenges to targeted and immunotherapy in digestive cancers and provide an outlook to innovative therapeutic developments that are expected to shape the management of these cancers in the future.

### Therapy of digestive cancers—current challenges

#### Tumor heterogeneity and plasticity

From the perspective of tumor biology, the heterogeneous response of primary tumors and metastases to anticancer therapies can be attributed to two main characteristics of cancers: tumor heterogeneity and plasticity. Tumor heterogeneity can occur at the genetic, epigenetic and environmental level which can all affect treatment response.^486^ Intratumoral genetic heterogeneity is a pervasive feature of most cancers, with tumor genome sequencing approaches showing subclonal populations in 95.1% of 1.705 tumors from 38 different cancer types.^487^ Genetic heterogeneity can also occur at the level of copy number alterations. For instance, while mutations are more similarly distributed in CRC, copy number variation was found to be the dominant genetic alteration that drives intratumoral heterogeneity in this cancer type.^488^ Interestingly, this intratumoral genetic diversity can also be increased by drug treatment, as CRC responds to inhibition of Ras signaling by increasing mutagenesis rate via downregulation of DNA repair genes and increased expression of error-prone Y family polymerases.^489^ Longitudinal sampling of CRC patients undergoing anti-EGFR therapy shows that many resistance-mediating mutations are already present at the time of diagnosis and that clinical drug resistance is significantly driven by polyclonal mechanisms involving multiple alterations.^490,491^ A major challenge is to monitor the genetic heterogeneity of cancers over time without invasive tumor biopsies. In this respect, several studies showed that drug resistance conferring mutations can be detected by sequencing of ctDNA, providing a diagnostic tool to capture the adaptive genetic landscape of cancers under therapy.^491,492^

Over the last few years, novel methods of cellular profiling, such as single-cell sequencing have provided novel insights into tumor heterogeneity that extends beyond genetics, thereby revealing a more granular landscape of tumor biology. It became evident that tumor plasticity is a prevalent feature of many cancer types. Tumor plasticity is the ability of cancer cells to adapt to environmental perturbations such as antineoplastic treatment without changing their genetic background.^493^ For instance, single-cell sequencing in melanoma revealed a transient variability in gene expression with a small population of cancer cells expressing high levels of drug-resistance genes. Upon drug treatment, epigenetic reprogramming in this cell population then leads to a persistence of this transcriptional state.^494^ Functional heterogeneity is also a pre-existing tumor feature in pancreatic cancer. Using clonal replicate xenograft models of pancreatic cancer, it was shown that tumors deriving from the same clonal population can show diverse responses to gemcitabine chemotherapy, which is caused by genetic and transcriptional diversification.^495^ Moreover, tumor-extrinsic factors can confer cellular heterogeneity and drug resistance. For instance, resistance to kinase inhibitors can be mediated by cues that are derived from paracrine secretion of tumors and the microenvironment, such as hepatocyte growth factor.^495^ The tumor microenvironment can also reshape fibroblasts which then promote extracellular matrix production and remodeling, leading to elevated integrin β1/FAK/Src signaling that rescues the antiproliferative effect of BRAF inhibitors in melanoma.^496^ However, many of these mechanisms were observed in preclinical murine cancer models, primarily of melanoma. In contrast, how tumor heterogeneity and plasticity shape the treatment response of gastrointestinal cancers in patients is largely unknown. Apart from the heterogeneous expression of HER2 in gastric cancer that requires multiple biopsies from different tumor locations, neither tumor heterogeneity nor plasticity are currently embedded in routine diagnostic or therapeutic concepts for digestive cancers.

#### Immune evasion of gastrointestinal cancers

Over the last years, immunotherapy has become a major pillar for the treatment of all metastatic digestive cancers. While immune checkpoint inhibitors are highly efficient in tumors with microsatellite instability, particularly CRC^465^, the clinical benefit is less pronounced in other cancer types of the digestive system. The underlying reasons for this heterogeneous response to immune checkpoint inhibitors have been a major focus of research. Mechanisms of immune evasion differ between digestive cancers, and can be classified into tumor intrinsic and extrinsic mechanisms. Intrinsic mechanisms include genetic alterations leading to the activation of oncogenic signaling pathways that mediate immune evasion. For instance, genetic activation of the Wnt pathway was strongly associated with T-cell exclusion in CRC.^497^ In a mouse model of HCC, genetic activation of beta-catenin, the main mediator of Wnt signaling, promoted immune escape via defective recruitment of dendritic cells.^498^ Furthermore, activating gene alterations of the Ras-MAPK pathway were also found to mediate immune evasion. For instance, *ERBB2/ERBB3* mutations induced the expression of PD-L1 in gallbladder cancer and suppressed T-cell-mediated cytotoxicity in vitro.^499^ In a mouse model of pancreatic cancer with different strengths of KRAS activation, mutant *KRAS* could induce immune escape by changing the composition of immune cells in the tumor.^500^ Similarly, in CRC, KRAS activation induced the expression of CXCL3 via IRF2, which triggered the infiltration of myeloid-derived suppressor cells into the adjacent microenvironment.^501^ Furthermore, a pan-cancer analysis revealed that loss of heterozygosity of HLA-I tends to eliminate the HLA allele which predominantly present neoepitopes.^502^ Interestingly, low levels of neoantigen presentation were widely observed in microsatellite-stable CRC, which however reduced correct priming of T-cells and thereby affected immunosurveillance.^503^ Tumor extrinsic mechanisms that confer immune escape comprise mostly autocrine and paracrine signals such as growth factors and chemokines that affect the innate and adaptive immune response.^504^ Hence the difference in response to immunotherapy might be attributed to the heterogeneous tumor microenvironments of solid cancers.^505^ For instance, high TGF-β levels in the tumor microenvironment of CRC liver metastases can create an immuno-repressive state, and blockage of TGF-β increases sensitivity of tumors to immune checkpoint blockade.^506^ TGF-β derived from tumor-associated monocytes could also affect the function of the innate immune system, including natural killer cells, in gastric cancer.^507^ Establishment of an immunosuppressive microenvironment via chemokines is another recurrent mechanism of immune escape in digestive cancers. For instance, CCL2 production by esophageal squamous cell cancers was associated with increased infiltration of tumor-associated macrophages. Similarly, HCC-derived CCL15 was shown to induce the infiltration of CCR1+/CD14+ monocytes with multiple immunosuppressive features.^508^ In gastric cancer, macrophages can secrete CXCL8 which increases their PD-L1 expression while hampering CD8+ T-cell function in the tumor itself.^509^ Changes in the metabolome of the tumor microenvironment can also affect immune cell function. Gastric cancer cells were found to overexpress the taurine transporter SLC6A6, thereby depleting taurin in the tumor microenvironment and induce taurine deficiency in CD8+ T cells, leading to ER stress and immune exhaustion.^510^ Finally, several studies have also revealed potential druggable immune targets for selective digestive cancers. For instance, in pancreatic cancer, immune response in neoantigen-specific CD8+ T cells is dysfunctional, and interference with the druggable CD155/TIGIT-axis could abolish this immune escape.^511^ Notably, multi-omics profiling and single-cell sequencing studies showed that extensive reshaping of the immune microenvironment is tumor-stage dependent, with different classes of immune cells affected at different stages.^512,513^ Hence, depending on the tumor stage, different immuno-oncological treatment strategies might be needed. In summary, with growing insights into the tumor-specific mechanisms of immune evasion, the next aim will be to target these mechanisms and interactions by combination therapies to overcome resistance to current immunotherapeutic strategies (see also selection of ongoing trials with immune checkpoint inhibitors in Table. 1, 2).

**Table 2:** Selection of ongoing clinical trials investigating novel targeted therapies directed against oncogenic signaling pathways in metastatic digestive cancers

Cancer	Stage	Treatment line	Target	Treatment	Phase	Identifier	Status
Gastric/GEJ adenocarcinoma / PDAC	IV or unresectable	1–3	CLDN18.2	A: AZD0901 (antibody-drug conjugate) B: AZD0901 + Chemotherapy	2	NCT06219941	Recruiting
CRC	IV / KRAS(G12C)	2	KRAS (G12C) + EGFR	A: MRTX849 + Cetuximab B: mFOLFOX6 / FOLFIRI	3	NCT04793958	Active, not recruiting
CCA	Relapsed, unresectable or metastatic cholangiocarcinoma; FGFR-altered, Chemotherapy or FGFR Inhibitor-Refractory	≥2	FGFR2	A: Tinengotinib B: Physician´s Choice	3	NCT05948475	Recruiting
CCA	Locally advanced unresectable or metastatic, HER2 positive	1	HER2 (bispecific antibody)	A: Zanidatamab + SOC (Gem/Cis ± PD-1/L1 inhibitor) B: SOC	3	NCT06282575	Recruiting
PDAC	advanced, recurrent, or metastatic pancreatic adenocarcinoma; pretreated	≥2	NECTIN-4	A: enfortumab vedotin	2	NCT05915351	Active

*GEJ* gastroesophageal junction, *EGFR* epidermal growth factor receptor, *CCA* cholangiocellular carcinoma, *CRC* colorectal cancer, *TKI* tyrosine kinase inhibitors, *CLDN18.2* claudin 18.2, *FGFR2* fibroblast growth factor receptor 2, HER2 human epidermal growth factor receptor 2

#### Translation of cancer genomics data into actionable targets

In recent decades, multiple genome sequencing projects have characterized the genetic landscape of almost all digestive cancers in unprecedented detail.^421,514,515^ This information has yielded a wealth of potentially actionable targets which were in part validated in tumor agnostic, prospective precision oncology trials such as the MOSCATO-01 or NCI-MATCH trial.^516,517^ Results of these studies showed that genomic profiling can result in clinical benefit for specific subgroups, with objective responses observed in 10-11% of all patients under investigation. However, data from these studies also clearly showed that only few targeted drugs exhibit clinical activity across multiple tumor types with the same molecular alteration (e.g. NTRK inhibitors in cancers with NTRK fusion or immune checkpoint inhibition in tumors with microsatellite instability), while the efficacy of most targeted therapies were influenced by the tissue background of the tumor.^518^ Prominent examples are the divergent effect of vemurafenib in *BRAF V600E* mutant melanoma versus CRC^519,520^, or sotorasib in *KRAS G12C* mutant NSCLC versus CRC.^474^ These observations indicate that the complexity of tumor biology and the interaction with the microenvironment requires clinical validation of actionable molecular alterations in a tissue-specific context. However, with the growing spectrum of targeted therapy options for solid cancers, precision oncology is being implemented into routine cancer care in a growing number of medical centers. Retrospective studies from these centers showed that exome and panel gene sequencing can already improve oncological outcome in real-world settings in subgroups of patients with digestive cancers.^521–523^ Despite this progress, wide-spread implementation of precision oncology for the management of cancers of the digestive tract remains challenging, requiring a multi-level effort, including a more equal access to genetic services, as well as infrastructures and resources to analyze and interpret genomic data.^524^

### Therapy of digestive cancers—future directions

Beyond the research progress in the treatment of specific cancer entities, which are described in the preceding chapters, there are also ongoing scientific efforts that might fundamentally reshape the therapeutic landscape of many cancers of the digestive system (see Tables 1 and 2 for current trials and Fig. 7 for future strategies). The following chapter concisely describes a selection of these therapeutic developments of which most are in early clinical testing (e.g., CAR-T cells, pan-KRAS inhibitors) in specific digestive cancers.

#### CAR-T cell therapy

Adoptive cell therapies utilizing T cells that express engineered chimeric antigen receptors (CARs) have shown remarkable success in treating hematologic malignancies.^525,526^ However, they failed to produce similar clinical efficiency in early trials in solid cancers, especially gastrointestinal tumors.^527^ The underlying reasons are manifold and include a limited number of targetable tumor-associated antigens, the heterogeneous expression of these antigens, and T-cell dysfunction caused by an immunosuppressive tumor microenvironment, amongst others.^528,529^ Despite these limitations, several tumor-associated antigens have been evaluated as targets for CAR-T cell therapy in cancers of the digestive system, some of these in early clinical trials. Trop2 is highly expressed on the cell surface of gastrointestinal tumors such as CRC or GC.^530,531^ Studies using Trop2/PD-L1 CAR-T cells in mouse models of gastric cancer showed reduced tumor growth upon intratumoral injection of the engineered T-cell.^532^ Also, as these CAR-T cells target both Trop2 and PD-L1, they produced higher levels of IFN-y and IL-2, thereby increasing the immune response.^532^ Another target for CAR-T cell therapy is HER2, as it is overexpressed in 30% of solid tumors. Preclinical studies with HER2-specific CAR-T cells demonstrated its potential for the treatment of gastrointestinal cancers, especially CRC.^533^ Phase I clinical trials using CAR-T cell therapy targeting HER2 as antigen in pancreatic and CCA showed feasibility and manageable adverse effects, but needs further evaluation regarding their clinical efficacy.^534^ Similarly, EGFR targeting CAR-T cell therapy was assessed in EGFR-positive CCA in a phase I trial, which demonstrated a favorable safety profile.^535^ CLDN18.2 is another highly expressed cell surface antigen in cancers of the digestive system, particularly GC. CT041 is a CLDN18.2-specific CAR-T therapy that was tested in a phase I study.^536^ Data from this trial showed promising oncological effects of CT041 with high disease control rates, but also very high frequencies of cytokine release syndromes as well as hematological toxicities.^536^ In HCC patients, the surface antigen GPC3 is overexpressed while it is almost not found on untransformed tissues. In a phase I study, HCC patients who progressed on prior therapy received a single infusion of C-CAR031, a GPC3-targeting CAR-T cell therapy that co-express a dominant negative TGF-b receptor II.^537^ The objective response rate was 50% in this small cohort of 22 patients. Despite the high frequency of hematological toxicities, these results highlight the potential of CAR-T cell therapy for hard-to-treat cancers. Another direction of research is to combine CAR-T therapy with other immune modulators, chemotherapies, or tumor vaccines. An ongoing clinical trial shows the potential of combining CAR-T cell therapy targeting CLDN6 and an RNA vaccine encoding CLDN6 that increases antigen presentation by antigen-presenting cells.^538^ Patients with solid tumors treated with this combination showed a promising ORR of 59% and longer CAR-T cell persistence upon vaccination.

#### Novel KRAS inhibitors

Oncogenic KRAS mutations are frequently detected in digestive cancers, predominantly in PDAC, CRC and CCA, and to a lesser extent also in ESCC and GC.^539^ Due to the dominant role of KRAS mutations in the oncogenesis of many digestive cancers, it has been considered as an outstanding, yet undruggable target for many decades.^540^ However, the seminal discovery of molecules that covalently bind to the cysteine residue of KRAS G12C, thereby reducing GDP/GTP exchange and impairing binding to Raf, has sparked the development of a new class of KRAS inhibitors.^541^ Over the last five years, clinical testing and approval of KRAS G12C-specific inhibitors accelerated after initial success in clinical trials with non-small cell lung cancer.^474^ However, monotherapy with KRAS G12C inhibitors (e.g., sotorasib and adagrasib) is less effective in CRC^474^, due to different tumor intrinsic compensatory mechanisms, for instance activation of upstream EGFR signaling.^474^ Hence, the combination of anti-EGFR antibodies with different KRAS G12C inhibitors showed superior oncological effects over monotherapy in CRC.^475^ Treatment with KRAS G12C inhibitors also demonstrated clinical benefit in other digestive cancers such as PDAC, but due to the overall low frequency of the G12C genotype in this cancer entity, only a few patients were included in the initial clinical trial.^404^ Hence, the current focus of research is the development and clinical testing of KRAS inhibitors for other or all genotypes. Non-covalent KRAS G12D inhibitors showed robust antineoplastic effects in preclinical models of PDAC^405,542^, and a phase I clinical trial in this cancer entity was initiated (NCT05737706). Tri-complex allele-specific KRAS inhibitors are another class of drugs that act as a molecular glue with cyclophilin A, and are able to overcome some of the resistance mechanisms of covalent KRAS G12C inhibitors.^543^ Preliminary data from phase I trials with these tri-complex inhibitors showed promising clinical effects in solid tumors.^544^ Another direction of research is the development of pan-KRAS inhibitors that can overcome the allele-specificity of the present inhibitors. RMC-6236 is a tri-complex pan-Ras inhibitor that is currently being tested in a phase I clinical trial in solid tumor.^545^ Preliminary results of this trial showed clinical benefits in pancreatic and non-small cell lung cancers. A different type of pan-KRAS inhibitor that was recently published is BI-2865, which binds exclusively to KRAS and not to other RAS proteins^546^, and is also under clinical investigation (NCT06056024). Major efforts are currently ongoing to combine KRAS inhibitors with other molecularly targeted drugs of the Ras-MAPK pathway, chemotherapy, and immune checkpoint inhibitors.^476^ It is therefore expected that KRAS inhibitors will reshape the therapeutic landscape of *KRAS* mutant cancers of the gastrointestinal tract in the upcoming years.

#### Novel targeted inhibitors of other oncogenic pathways

Beyond the Ras pathway, there is considerably less progress in the development of drugs that target other signaling pathways that are altered in many gastrointestinal tumors (see also selection of ongoing trials in Table 2). Aberrant Wnt activation is highly prevalent in most cancers of the digestive system including colorectal and liver cancers.^547^ So far, multiple efforts to target Wnt signaling in solid cancers have not met the expectations.^548^ Two classes of Wnt inhibitors have been most extensively investigated in clinical trials: porcupine inhibitors and DKK1 antagonists. Porcupine inhibitors reduce the palmitoylation of Wnt ligand protein, which is essentially required for their secretion. A phase I clinical trial investigated the porcupine inhibitor WNT974 in a cohort of solid cancers, including CRC and pancreatic cancers.^549^ There were no tumor responses observed despite sufficient on-target inhibitory effect. In a more recent trial with porcupine inhibitors, preliminary results show that patients with CRC that harbor RSPO3 fusions, which renders tumor growth dependent on Wnt ligands, might benefit from a combination of porcupine inhibition and immune checkpoint blockade.^550^ DKK1 is an antagonist of the Wnt pathway that can be targeted by the antibody DKN-01. Early-phase clinical trials were performed in esophagogastric adenocarcinoma and colorectal cancer in combination with chemotherapy, anti-angiogenic antibodies, or immune checkpoint inhibitors. Preliminary results from these studies indicate clinical activity of DKN-01 in colorectal^551^ and GEJ/gastric adenocarcinoma^552^ that will be followed up in ongoing trials. The fibroblast growth factor receptor (FGFR) signaling pathway plays a pleiotropic role in development, cell proliferation, differentiation, and angiogenesis.^553^ Gastric cancers and cholangiocellular carcinoma harbor activating mutations of FGFR receptors or gene fusions, or overexpress specific receptor isoforms, such as FGFR2b. While inhibitors of FGF fusion proteins are highly efficient in cholangiocellular carcinoma, no clinical benefit was observed in CRC.^554^ Recently, bemarituzumab, an antibody targeting FGFR2b, was shown to improve survival in combination with chemotherapy in a phase II trial in patients with gastric or GEJ adenocarcinoma with high expression of FGFR2.^555^ Together, these recent developments in targeted therapies of different oncogenic pathways indicate that for selected cohorts of cancer patients, additional molecularly targeted therapies either alone or in combination with immune therapies, will potentially generate additional clinical benefit.

#### Novel antibody-drug conjugates

Antibody-drug conjugates (ADCs) are an established class of drugs that combines the specificity of antibodies with the potency of cytotoxic agents. Since 2013, the FDA has approved four ADCs for the treatment of solid cancers, mostly of gynecological origin.^556^ The first approved ADC for the treatment of digestive tract cancers is trastuzumab deruxtecan, which showed a significant survival benefit in patients with pretreated HER2-positive gastric or GEJ adenocarcinoma.^100^ Following these results, the activity of trastuzumab deruxtecan was also shown in HER2-positive CRC in the phase II DESTINY-CRC01 trial^472^ and in HER2-positive CCA.^557^ Currently, there are several directions that are pursued to expand the therapeutic application of ADCs in digestive cancers. One direction is to develop and test ADCs that act against other established therapeutic targets in this group of cancers, such as CLDN18.2. A prominent example is CMG901, an anti-CLDN18.2 ADC that uses monomethyl auristatin E (MMAE) as a chemotherapeutic payload. In a recent phase Ia clinical trial including gastric/GEJ adenocarcinoma and pancreatic cancers, CMG901 showed an ORR of 75% in patients with CLDN18.2 positive, pretreated gastric/GEJ adenocarcinoma.^558^ Another strategy is to apply ADCs that were developed and approved for other cancer entities, such as sacituzumab govitecan for Trop2-positive breast and urothelial cancers, in gastrointestinal tumors that share a high expression of the same target protein^521,522^. Despite promising antineoplastic effects in preclinical models of colorectal and pancreatic cancer^559^, no clinical benefit has yet been reported for sacituzumab govitecan or other Trop2 targeting ADCs in digestive tract cancers.^560^ Finally, a major goal is to develop ADCs that are directed against novel, highly expressed cell surface proteins in digestive cancers. Several preclinical studies suggest that CDH17, a cell adhesion protein that is overexpressed in many digestive cancers, is a potentially suitable target. ADCs based on anti-CDH17 antibodies linked to MMAE showed potent anti-tumor activity in xenograft models of PDAC and CRC.^561,562^ Other targets that showed significant tumor response in preclinical models when targeted by ADCs include GPR56 in CRC or GPC-1 in pancreatic cancer.^563,564^ Whether these ADCs can confirm their efficacy in clinical trials is an open question. Further efforts undertaken to improve the overall efficacy of ADCs include optimization of the chemotherapeutic payload and linker technologies in a tumor-specific context, the use of modified antibodies, and improvement of biomarker-based patient selection to minimize adverse effects.^565^

#### Proteolysis-targeting chimeras (PROTACs)

Currently, most targeted therapeutic approaches in digestive cancers rely either on cell surface proteins that are accessible to antibodies or functional domains of intracellular proteins that can be inhibited by small molecules. Due to this limitation, many key oncogenes that act as transcriptional (co-)factors, such as MYC or beta-catenin, are not accessible to established therapeutic concepts, demanding other approaches. Proteolysis-targeting chimeras (PROTACs) are heterobifunctional compounds that are composed of two ligands, one binding a protein of interest and another binding an E3 ubiquitin ligase, connected by a chemical linker. PROTACs induce degradation of target proteins by bringing it into close proximity to the E3 ubiquitin ligase, which results in its poly-ubiquitination.^566^ In 2020, results of the first proof-of-principle clinical trials confirmed that the PROTACs ARV-110 and ARV-471 could reduce the levels of androgen and estrogen receptors in patients with prostate or breast cancer, respectively, which was associated with clinical benefit and manageable drug toxicity.^567,568^ These results have accelerated the development of other PROTACs and their validation in clinical trials for different tumor entities, including solid cancers and targets that are relevant for digestive cancers such as BCL-XL, BRD4, or STAT3.^569^ Until now, clinical trials testing PROTACs specifically in digestive cancers have not been initiated.^570^ However, there is growing evidence from preclinical models that PROTACs could be effective in digestive cancers, targeting oncoproteins that are otherwise not accessible to pharmacotherapy. For instance, a peptide-based PROTAC could stably degrade beta-catenin and reduce Wnt signaling in organoids and mouse models of CRC, leading to tumor regression.^571^ With the advent of final clinical results from several phase II trials with the first PROTACs, and the ongoing advancement of PROTAC technology, it is expected that this novel class of drugs will also reshape the treatment of digestive cancers.

### Concluding remarks

Cancers of the digestive system present a major disease burden with almost all tumors having a poor prognosis in the metastatic stage. Over the last decades, significant progress has been made in deciphering the genetic basis of digestive cancers through large-scale genome sequencing efforts, providing insights into genetic drivers of cancer initiation and progression at unprecedented granularity. Furthermore, molecular interactions of digestive cancers with their microenvironment have been gradually deciphered, resulting in a better understanding of mechanisms that drive immune evasion and the discovery of molecular targets for interventions. This knowledge has led to the development and wide-spread introduction of immunotherapies as a main pillar of anticancer therapy, which improved overall survival for patients with cancers of the digestive system. The potential of immunotherapy is yet unfolding, with novel immune targets such as LAG3 or VISTA being evaluated, and CAR-T cell therapy showing the first promising results in gastric and hepatocellular carcinomas. Furthermore, novel targeted therapies have largely expanded the therapeutic toolbox for selected cancer types such as CRC, CCA and GC. Most targeted therapies are directed against the Ras-MAPK signaling pathway, as activating mutations of RAS oncogenes are a hallmark of many digestive cancers. With the introduction of genotype specific KRAS inhibitors and the advent of pan-KRAS inhibitors, it is expected that more patients with digestive cancers will benefit from targeted therapies. This precision oncology approach will be complemented by novel antibody classes such as bispecific antibodies and antibody-drug conjugates that target tumors with abundant expression of specific cell surface proteins, such as CLD18.2. Further molecular characterization of digestive cancers will yield additional cell surface targets in the future, thereby expanding the therapeutic spectrum of antibodies. With progress in these different fields of medical therapy, it will be a major challenge to identify optimal combinations of immuno-, targeted and chemotherapies, and to discover biomarkers that predict response to these combinations. Finally, advances in other fields of cancer management, such as radiation therapy and surgery, will also reshape the treatment of cancers of the digestive system. Together, these developments will result in a more complex and multidisciplinary management of digestive cancers, leading to better oncological outcomes in the future.
